# Cardiovascular Risk According to Body Mass Index in Women of Reproductive Age With Polycystic Ovary Syndrome: A Systematic Review and Meta-Analysis

**DOI:** 10.3389/fcvm.2022.822079

**Published:** 2022-02-16

**Authors:** Chenchen Zhuang, Xufei Luo, Wenjuan Wang, Runmin Sun, Miaomiao Qi, Jing Yu

**Affiliations:** ^1^Hypertension Center, Lanzhou University Second Hospital, Lanzhou, China; ^2^School of Public Health, Lanzhou University, Lanzhou, China

**Keywords:** polycystic ovary syndrome, cardiovascular risk, reproductive-age, meta-analysis, body mass index

## Abstract

**Background:**

Polycystic ovary syndrome (PCOS) is a heterogeneous condition that affects women of reproductive age. The association between PCOS and cardiovascular risk according to body mass index (BMI) categories is unclear.

**Objective:**

We evaluated the association between cardiovascular risk according to BMI categories and PCOS in women of reproductive age.

**Methods:**

A literature search was conducted in the EMBASE, MEDLINE, Cochrane Library, and PubMed databases from their inception to 9 September, 2021. Observational cross-sectional, retrospective, and prospective controlled studies were included. The main analyses examined the relationship between cardiovascular risks (i.e., blood pressure and lipid levels) and BMI in women of reproductive age with PCOS.

**Results:**

Thirty-eight studies, with a total of 6,078 subjects, were included in this metaanalysis. Systolic blood pressure (SBP) and diastolic blood pressure (DBP) were higher in women of reproductive age with PCOS. Lower high-density lipoprotein (HDL)-cholesterol [SMD (95% CI): −0.21 (−0.35, −0.08), *p* = 0.002], higher triglycerides [SMD (95% CI): 0.38 (0.29, 0.48), *p* < 0.001], higher low-density lipoprotein (LDL)-cholesterol [SMD (95% CI): 0.29 (0.20, 0.39), *p* < 0.001], higher nonHDL-cholesterol [SMD (95% CI): 0.42 (0.31, 0.52), *p* < 0.001] and waist-to-hip ratio (WHR) [MD (95% CI): 0.03 (0.02, 0.04), *p* < 0.001] were seen in women of reproductive age with PCOS. In addition, the subgroup analysis revealed that systolic BP and HDL-cholesterol increased at BMI < 25 kg/m^2^ and BMI 25–30 kg/m^2^. Diastolic BP increased at BMI 25–30 kg/m^2^. Triglycerides, LDL-cholesterol, nonHDL-cholesterol, and WHR increased in all BMI categories.

**Conclusions:**

PCOS is associated with cardiovascular risk. Lipid levels and BP increased in women of reproductive age with PCOS, regardless of BMI.

**Systematic Review Registration:**

Open Science Framework (10.17605/OSF.IO/92NBY).

## Introduction

Polycystic ovary syndrome (PCOS) is one of the most common female endocrinopathies and is a highly prevalent disorder that affects ~7–14% of women of reproductive age (1, 2). The clinical manifestations of PCOS are heterogeneous; however, the hallmarks of the syndrome are anovulation, insulin resistance, and androgen excess. Furthermore, PCOS leads to adverse metabolic sequelae, including high blood pressure, dyslipidemia, and obesity, all of which individually confer a cardiovascular risk. Each of these features promotes cardiovascular risk in this population. The American Society for Reproductive Medicine Practice Committee reported that cardiovascular risk was increased in women with PCOS (3). Recent studies have shown that cardiovascular risk factors are more frequent among women with PCOS than among women without PCOS (4, 5). Apart from the deleterious effects of PCOS *per se*, individual risk markers including blood pressure and lipid profile are important mediators of further cardiovascular outcomes. Therefore, PCOS may represent an important key to lipid and blood pressure alterations starting at the age of reproduction.

The effect of PCOS on obesity and that of obesity on PCOS is complex. However, it is agreed that the prevalence of increased body mass index (BMI) is higher in women with PCOS (6, 7). A previous metaanalysis showed that obesity was more prevalent in women with PCOS than in women without PCOS (8). PCOS occurs both in obese and lean women. However, studies of cardiovascular risk and PCOS have not distinguished the effects of BMI from those of PCOS. In addition, lipid profiles may differ in their association with blood pressure and PCOS. However, there is little consensus that the possible increase in cardiovascular risk among women with PCOS is merely related to obesity. Based on BMI, our metaanalysis classified patients as underweight (<18.5 kg/m^2^), normal weight (18.5–25 kg/m^2^), overweight (25–30 kg/m^2^), and obese (≥30 kg/m^2^) (9). These standard categories have been increasingly used in published studies of BMI and PCOS, but the literature has not been systematically reviewed.

The present metaanalysis examined the relationship between cardiovascular risk (i.e., blood pressure and lipids) and BMI in women of reproductive age with PCOS. To reduce biases that may appear in all metaanalyses, only BMI-matched studies with a sufficient number of subjects were included.

## Methods

The present study was approved by the Ethics Committee Board of Lanzhou University Second Hospital (D2019-098) and conducted in accordance with the Preferred Reporting Items for Systematic Reviews and Meta-Analyses (PRISMA) (10). The protocol was prospectively registered with Open Science Framework (10.17605/OSF.IO/92NBY).

### Search Strategy

Two independent authors (CC Zhuang and XF Luo) searched the Cochrane Library, EMBASE, and MEDLINE databases from inception to 9 September 2021 for full-text articles without any language restriction. The medical subject headings (MeSHs) and keywords included polycystic ovary syndrome, Stein Leventhal, cardiovascular risk, blood pressure, hypertension, lipoprotein, dyslipidemia, and hyperlipidemia (see Table 1 for the detailed search strategies). For all identified studies, a manual search was conducted of their references and review articles to locate additional relevant studies.

**Table 1:** Search strategies.

**PubMed**
**Polycystic ovary syndrome** #1 (Polycystic Ovary Syndrome [mh]) OR (Ovary Syndrome, Polycystic [ti/ab]) OR (Syndrome, Polycystic Ovary [ti/ab]) OR (Stein-Leventhal Syndrome [ti/ab]) OR (Stein Leventhal Syndrome [ti/ab]) OR (Syndrome, Stein-Leventhal [ti/ab]) OR (Sclerocystic Ovarian Degeneration [ti/ab]) OR (Ovarian Degeneration, Sclerocystic [ti/ab]) OR (Sclerocystic Ovary Syndrome [ti/ab]) OR (Polycystic Ovarian Syndrome [ti/ab]) OR (Ovarian Syndrome, Polycystic [ti/ab]) OR (Polycystic Ovary Syndrome 1 [ti/ab]) OR (Sclerocystic Ovaries [ti/ab]) OR (Ovary, Sclerocystic [ti/ab]) OR (Sclerocystic Ovary [ti/ab]) **Cardiovascular risk** #2 (Cardiovascular Risk [mh]) OR (Cardiovascular Risk Factors [ti/ab]) OR (Cardiovascular Risk Factor [ti/ab]) OR (Factor, Cardiovascular Risk [ti/ab]) OR (Risk Factor, Cardiovascular [ti/ab]) OR (Risk Factors for Heart Disease [ti/ab]) OR (Risk Factors for Cardiovascular Disease [ti/ab]) OR (Cardiovascular Risk Score [ti/ab]) OR (Cardiovascular Risk Scores [ti/ab]) OR (Risk Score, Cardiovascular [ti/ab]) OR (Score, Cardiovascular Risk [ti/ab]) OR (Cardiovascular Risk [ti/ab]) OR (Cardiovascular Risks [ti/ab]) OR (Risk, Cardiovascular [ti/ab]) OR (Residual Cardiovascular Risk [ti/ab]) OR (Cardiovascular Risk, Residual [ti/ab]) OR (Residual Cardiovascular Risks [ti/ab]) OR (Risk, Residual Cardiovascular [ti/ab]) #3 (Lipid Level [mh]) OR (Accumulation Product, Lipid [ti/ab]) OR (Accumulation Products, Lipid [ti/ab]) OR (Lipid Accumulation Products [ti/ab]) OR (Product, Lipid Accumulation [ti/ab]) OR (Products, Lipid Accumulation [ti/ab]) #4 (Hypertension [mh]) OR (Blood Pressure, High [ti/ab]) OR (Blood Pressures, High [ti/ab]) OR (High Blood Pressure [ti/ab]) OR (High Blood Pressures [ti/ab]) **Study design** #5 (Cross-sectional studies [Mesh]) OR (Cross-sectional studies [All fields]) OR (Cross-sectional [All fields]) OR (Prevalence Studies [All fields]) OR (Prevalence Study [All fields]) OR (Survey [All fields]) OR (Prevalence [All fields]) #6 OR #2 OR #3 OR #4 #7 #1 AND #5 AND #6
**Cochrane library**
#1 MeSH descriptor: [Polycystic Ovary Syndrome] explode all trees #2 'ovary polycystic disease':ab,ti,kw #3 'Polycystic Ovarian disease':ab,ti,kw #4 'PCOS':ab,ti,kw #5 'polycystic ovarian syndrome':ab,ti,kw #6 'polycystic ovary syndrome':ab,ti,kw #7 OR/#1-#6 #8 MeSH descriptor: [Heart Disease Risk Factors] explode all trees #9 MeSH descriptor: [Lipids] explode all trees #10 MeSH descriptor: [Hypertension] explode all trees #11 'cardiovascular risk':ab,ti,kw #12 'lipid level':ab,ti,kw #13 'lipid blood level':ab,ti,kw #14 'hypertension':ab,ti,kw #15 'Blood Pressure':ab,ti,kw #16 'Lipid Accumulation':ab,ti,kw #17 OR/#8-#16 #18 MeSH descriptor: [Cohort Studies] explode all trees #19 MeSH descriptor: [Case-Control Studies] explode all trees #20 MeSH descriptor: [Cross-Sectional Studies] explode all trees #21 'cross-sectional study':ab,ti,kw #22 'case control study':ab,ti,kw #23 'cohort analysis':ab,ti,kw #24 'cohort study':ab,ti,kw #25 'Prevalence Study':ab,ti,kw #26 'Survey':ab,ti,kw #27 OR/#18-#26 #28 #7 AND #17 AND #27
**Embase**
#1 'ovary polycystic disease'/exp
#2 'ovary polycystic disease':ab,ti,kw #3 'Polycystic Ovarian disease':ab,ti,kw #4 'PCOS':ab,ti,kw #5 'polycystic ovarian syndrome':ab,ti,kw #6 'polycystic ovary syndrome':ab,ti,kw #7 OR/#1-#6 #8 'cardiovascular risk'/exp #9 'cardiovascular risk factor'/exp #10 'lipid level'/exp #11 'lipid blood level'/exp #12 'hypertension'/exp #13 'cardiovascular risk':ab,ti,kw #14 'lipid level':ab,ti,kw #15 'lipid blood level':ab,ti,kw #16 'hypertension':ab,ti,kw #17 'Blood Pressure':ab,ti,kw #18 'Lipid Accumulation':ab,ti,kw #19 OR/#10-#18 #20 'cross-sectional study'/exp #21 'case control study'/exp #22 'cohort analysis'/exp #23 'survey'/exp #24 'cross-sectional study':ab,ti,kw #25 'case control study':ab,ti,kw #26 'cohort analysis':ab,ti,kw #27 'cohort study':ab,ti,kw #28 'Prevalence Study':ab,ti,kw #29 'Survey':ab,ti,kw #30 OR/#20-#29 #31 #7 AND #19 AND #30

### Study Selection and Criteria

Two reviewers (CC Zhuang and XF Luo) independently selected the articles by title, abstract and then full-text. The selection criteria for the retrieved articles in our metaanalysis were as follows: (1) the studies were observational cross-sectional, retrospective, and prospective controlled; (2) the subjects were women of reproductive age with PCOS (18–49 years old), diagnosed by the Rotterdam criteria or that of the National Institutes of Health; (3) the subjects did not have comorbidities; (4) there was no evidence of androgen-secreting tumors, congenital adrenal hyperplasia, or medication that altered androgen metabolism or lipids; and (5) each article had to conduct BMI matching or the equal BMI (mean). Each study had to have evaluated ≥30 adult women with PCOS that were ≥18 years old but ≤ 45 years old (to avoid the perimenopausal transition). Control subjects without PCOS of the same age range had to be included. The exclusion criteria of the metaanalysis were as follows: (1) studies that were case-only; (2) studies with incomplete data; (3) articles that were metaanalyses, letters, reviews, or editorial articles. Non-patient community control studies were excluded, as PCOS occurs infrequently and some phenotypic elements of PCOS can occur in regularly menstruating women. The selected data were screened based on the search terms and the inclusion and exclusion criteria, and a PRISMA flow diagram was used to describe the stepwise article selection process in detail.

### Data Extraction

Authors of studies eligible for inclusion in the present metaanalysis were invited to join our study and share their data. The following data were extracted from the included studies: (1) name of the first author, (2) publication year, (3) type of study, (4) number of participants, (5) patient characteristics, (6) WHR, (7) androgen levels, and (8) patient outcomes. The outcomes included BMI, LDL-C or triglycerides (TG), low HDL-C, high SBP, DBP), and WHR. When not reported in the individual study, nonHDL-C was calculated as the total cholesterol minus HDL-C. Data extraction was independently performed by three investigators (CC Zhuang, XF Luo, and WJ wang). Disagreements were resolved through discussion.

### Quality of the Included Studies

The quality of the included studies was assessed by the Newcastle–Ottawa Scale (NOS), a validated scale for metaanalyses of observational studies (11, 12). The proposed scale was further adapted to the outcome of interest of this metaanalysis, and the items were divided into three domains [selection (representativeness of the sample, sample size, and non-responders), comparability (confounding factors) and outcome (blinding and calibration of the examiners); details are provided in Table 2]. The scale was also used to analyze cohorts and case-controls. We scored (maximum, nine points) the following items relevant to the risk of bias in non-randomized cohort studies: representativeness of the exposed cohort, adequate selection of controls, adequate definition of the outcome, adequacy of follow-up, and comparability of exposed and non-exposed women (two points). Studies with a group of comparisons were arbitrarily defined as having a high risk of bias if they scored between zero and three, moderate risk if they scored between four and six, and low risk if they scored between seven and nine.

**Table 2:** Characteristics of included studies in the meta-analysis.

**Author/Year**	**Place of study**	**Type of study**	**PCOS vs. control (*n*)**	**Participants age**	**BMI**	**WHR**	**Androgen level**	**Outcomes**
Akram et al. (13)	Pakistan	Retrospective	50 vs. 50	20-39 years	PCOS: 23.3 ± 0.67 kg/m^2^; Control: 21.8 ± 1.02 kg/m^2^	-	-	HDL-C, LDL-C, TG, nonHDL-C
Adali et al. (14)	Turkey	Prospective	26 vs. 25	PCOS: 24.73 ± 2.91 years; Control: 25.04 ± 2.26 years	PCOS: BMI <25 kg/m^2^; Control: BMI <25 kg/m^2^	PCOS: 0.74 ± 0.05; Control: 0.73 ± 0.04	-	HDL-C, LDL-C, TG, SBP, DBP, nonHDL-C
Alexandraki et al. (15)	Greece	Cross-section	27 vs. 27	PCOS: 25.41 ± 0.8 years; Control: 27.33 ± 0.83 years	PCOS: 25.41 ± 0.80 kg/m^2^; Control: 25.05 ± 1.19 kg/m^2^	PCOS: 0.78 ± 0.01; Control: 0.75 ± 0.01	PCOS: 10.85 ± 0.76 nmol/L; Control: 5.37 ± 0.38 nmol/L	HDL-C, LDL-C, TG, nonHDL-C
Arikan et al. (16)	Turkey	Prospective	39 vs. 30	PCOS: 22.82 ± 5.53 years; Control: 24.64 ± 4.22 years	PCOS: 21.48 ± 6.50 kg/m^2^; Control: 20.90 ± 6.04 kg/m^2^	-	PCOS: 2.98 ± 1.31 ng/ml; Control: 1.37 ± 0.89 ng/ml	HDL-C, LDL-C, TG, nonHDL-C
Berneis et al. (17)	Italy	Cross-section	30 vs. 24	PCOS: 25.1 ± 4.2 years; Control: 25.5 ± 3 years	PCOS: 28.4 ± 5.8 kg/m^2^; Control: 28 ± 4.4 kg/m^2^	-	-	HDL-C, LDL-C, TG, nonHDL-C
Cascella et al. (18)	Italy	Prospective	50 vs. 50	PCOS: 21.9 ± 2.7 years; Control: 22.2 ± 2.8 years	PCOS: 24.6 ± 2.5 kg/m^2^; Control: 24.4 ± 2.8 kg/m^2^	PCOS: 0.86 ± 0.1; Control: 0.83 ± 0.1	PCOS: 5.1 ± 0.7 nmol/L; Control: 1.4 ± 0.6 nmol/L	HDL-C, LDL-C, TG, SBP, DBP, nonHDL-C
Calzada et al. (19)	Spain	Retrospective	125 vs. 169	PCOS: 28.0 ± 5.0 years; Control: 30.0 ± 6.0 years	PCOS: 25.7 ± 7.1 kg/m^2^; Control: 22.3 ± 3.1 kg/m^2^	-	PCOS: 2.73 ± 1.35 ng/ml; Control: 2.17 ± 1.05 ng/ml	HDL-C, LDL-C, TG, SBP, DBP, nonHDL-C
Cetinkalp et al. (20)	Turkey	Prospective	129 vs. 91	PCOS: 24.58 ±4.61 years; Control: 25.48 ± 3.38 years	PCOS: 24.47 ± 4.64 kg/m^2^; Control: 24.2 ± 3.31 kg/m^2^	-	-	HDL-C, LDL-C, TG, nonHDL-C
Cheng et al. (21)	China	Prospective	103 vs. 96	PCOS: 26 ± 4 years; Control: 26 ± 2 years	PCOS: 24.2 ± 5.3 kg/m^2^; Control: 20.5 ± 2.7 kg/m^2^	PCOS: 0.9 ± 0.3; Control: 0.8 ± 0.1	-	HDL-C, LDL-C, TG, SBP, DBP, nonHDL-C
Cussons et al. (22)	Australia	Cross-section	19 vs. 19	PCOS: 30.4 ± 5.54 years; Control: 34.44 ± 7.8 years	PCOS: 24.1 ± 2.9 kg/m^2^; Control: 22.9 ± 3.2 kg/m^2^	PCOS: 0.8 ± 0.1; Control: 0.8 ± 0.6	PCOS: 10.89 ± 3.99 nmol/L; Control: 8.54 ± 2.41 nmol/L	HDL-C, LDL-C, TG, SBP, DBP, nonHDL-C
Diamanti-Kandarakis et al. (23)	Greece	Prospective	25 vs. 25	PCOS: 25.64 ± 0.86 years; Control: 27.52 ± 1.02 years	PCOS: 29.08 ± 1.43 kg/m^2^; Control: 26.22 ± 1.16 kg/m^2^	PCOS: 0.79 ± 0.01; Control: 0.75 ± 0.01	-	HDL-C, SBP, DBP, nonHDL-C
El-Kannishy et al. (24)	Egypt	Cross-section	14 vs. 10	PCOS: 25.2 ± 3.6 years; Control: 24.4 ± 4.07 years	PCOS: 22.8 ± 2.1 kg/m^2^; Control: 21.9 ± 2.97 kg/m^2^	-	-	HDL-C, LDL-C, TG, nonHDL-C
Erdogan et al. (25)	USA	Retrospective	68 vs. 26	PCOS: 24.27 ± 5.44 years; Control: 26.41 ± 5.65 years	PCOS: 24.41 ± 5.43 kg/m^2^; Control: 23.35 ± 5.04 kg/m^2^	-	-	HDL-C, LDL-C, TG, nonHDL-C
Erdogan et al. (26)	USA	Retrospective	88 vs. 119	PCOS: 24.07 ± 1.32 years; Control: 25.01 ± 2.05 years	PCOS: 24.38 ± 4.13 kg/m^2^; Control: 23.47 ± 4.12 kg/m^2^	-	-	HDL-C, LDL-C, TG, nonHDL-C
Glintborg et al. (27)	Denmark	Prospective	30 vs. 14	PCOS: 32.3 ± 7.9 years; Control: 34.3 ± 12.4 years	PCOS: 33.5 ± 4.2 kg/m^2^; Control: 32.8 ± 7.1 kg/m^2^	-	-	TG
González et al. (28)	USA	Cross-section	Lean: 10 vs. 10 Obese: 9 vs. 9	Lean: PCOS: 27 ± 1 years; Control: 29 ± 2 years; Obese: PCOS: 28 ± 2 years; Control: 32 ± 2 years	Lean: PCOS: 22.5 ± 0.6 kg/m^2^; Control: 34.4 ± 0.9 kg/m^2^; Obese: PCOS: 37.0 ± 6.9 kg/m^2^; Control: 34.1 ± 0.7 kg/m^2^	-	Lean: PCOS: 4.1 ± 0.4 ng/ml; Control: 1.8 ± 0.8 ng/ml; Obese: PCOS: 3.8 ± 0.3 ng/ml; Control: 2.0 ± 0.2 ng/ml	HDL-C, LDL-C, TG, SBP, DBP, nonHDL-C
Kargili et al. (29)	Turkery	Cross-section	168 vs. 52	PCOS: 25.7 ± 5.5 years; Control: 26.1 ± 5.4 years	PCOS: 26.8 ± 3.4 kg/m^2^; Control: 25.4 ± 2.8 kg/m^2^	-	-	HDL-C, SBP, DBP, nonHDL-C
Ketel et al. (30)	Netherlands	Cross-section	Lean: 22 vs. 17 Obese: 18 vs. 13	Lean: PCOS: 28.6 ± 4.5 years; Control: 27.7 ± 5.3 years; Obese: PCOS: 30.3 ± 4.2 years; Control: 28.6 ± 5.3 years	Lean: PCOS: 22.0 ± 2.2 kg/m^2^; Control: 22.2 ± 1.7 kg/m^2^; Obese: PCOS: 36.2 ± 5.9 kg/m^2^; Control: 40.5 ± 7.0 kg/m^2^	Lean: PCOS: 0.78 ± 0.05; Control: 0.76 ± 0.03; Obese: PCOS: 0.84 ± 0.05; Control: 0.80 ± 0.01	Lean: PCOS: 7.0 ± 1.8 nmol/L; Control: 4.8 ± 1.2 nmol/L; Obese: PCOS: 7.7 ± 2.4 nmol/L; Control: 4.9 ± 1.7 nmol/L	HDL-C, LDL-C, TG, SBP, DBP, nonHDL-C
Legro et al. (31)	USA	Cross-section	Lean: 42 vs. 27 Obese: 153 vs. 35	Lean: PCOS: 25 ± 65 years; Control: 29 ± 7 years; Obese: PCOS: 28 ± 5 years; Control: 32 ± 7 years	Lean: PCOS: 23.1 ± 2.4 kg/m^2^; Control: 23.0 ± 1.8 kg/m^2^; Obese: PCOS: 37.0 ± 6.9 kg/m^2^; Control: 37.7 ± 6.4 kg/m^2^	Lean: PCOS: 0.76 ± 0.07; Control: 0.75 ± 0.06; Obese: PCOS: 0.85 ± 0.10; Control: 0.79 ± 0.06	Lean: PCOS: 2553 ± 1367 ng/ml; Control: 1628 ± 734 ng/ml; Obese: PCOS: 2476 ± 1140 ng/ml; Control: 1533 ± 720 ng/ml^*^	HDL-C, LDL-C, TG, SBP, DBP, nonHDL-C
Liang et al. (32)	Taiwan	Prospective	Lean: 110 vs. 50 Obese: 110 vs. 20	Lean: PCOS: 26.8 ± 5.1 years; Control: 28.1 ± 4.2 years Obese: PCOS: 27.0 ± 6.4 years; Control: 29.0 ± 5.1 years	Lean: PCOS: 20.6 ± 2.0 kg/m^2^; Control: 20.4 ± 2.0 kg/m^2^; Obese: PCOS: 31.1 ± 3.9 kg/m^2^; Control: 30.4 ± 3.7 kg/m^2^	Lean: PCOS: 0.79 ± 0.06; Control: 0.82 ± 0.14; Obese: PCOS: 0.89 ± 0.08; Control: 0.85 ± 0.08	-	HDL-C, LDL-C, TG, nonHDL-C
Long et al. (33)	China	Cross-section	387 vs. 150	PCOS: 27.0 ± 4.5 years; Control: 25.3 ± 2.2 years	PCOS: 25.4 ± 4.6 kg/m^2^; Control: 20.7 ± 2.6 kg/m^2^	PCOS: 0.86 ± 0.06; Control: 0.80 ± 0.07	-	HDL-C, LDL-C, TG, SBP, DBP, nonHDL-C
Luque-Ramírez et al. (34)	Spain	Cross-section	40 vs. 20	Lean: PCOS: 23.0 ± 5.4 years; Control: 24.8 ± 6.0 years; Overweight: PCOS: 23.6 ± 4.6 years; Control: 29.3 ± 10.3 years; Obese: PCOS: 26.3 ± 6.7 years; Control: 28.5 ± 5.8 years	Lean: PCOS: 22.2 ± 2.0 kg/m^2^; Control: 21.3 ± 1.3 kg/m^2^; Overweight: PCOS: 27.5 ± 1.8 kg/m^2^; Control: 27.4 ± 1.5 kg/m^2^; Obese: PCOS: 35.8 ± 3.9 kg/m^2^; Control: 35.5 ± 3.2 kg/m^2^	Lean: PCOS: 0.73 ± 0.06; Control: 0.73 ± 0.06; Overweight: PCOS: 0.79 ± 0.07; Control: 0.79 ± 0.04; Obese: PCOS: 0.88 ± 0.09; Control: 0.83 ± 0.08	PCOS: 12.7 ± 3.6 nmol/L; Control: 7.2 ± 2.2 nmol/L	HDL-C, LDL-C, TG, SBP, DBP, nonHDL-C
Macut et al. (35)	Serbia	Prospective	75 vs. 51	PCOS: 23.1 ± 5.1 years; Control: 24.6 ± 4.1 years	PCOS: 24.9 ± 4.7 kg/m^2^; Control: 23.7 ± 4.0 kg/m^2^	PCOS: 0.79 ± 0.06; Control: 0.77 ± 0.05	-	HDL-C, LDL-C, TG, nonHDL-C
Meyer et al. (36)	Australia	Retrospective	100 vs. 20	PCOS: 32.7 ± 1.8 years; Control: 33.2 ± 2.3 years	PCOS: 37.3 ± 2.43 kg/m^2^; Control: 36.7 ± 1.28 kg/m^2^	PCOS: 0.86 ± 0.01; Control: 0.84 ± 0.02	PCOS: 4.9 ± 0.3 mmol/L; Control: 3.6 ± 0.4 mmol/L^*^	HDL-C, LDL-C, TG, SBP, DBP, nonHDL-C
Moran et al. (37)	Australia	Cross-section	80 vs. 27	PCOS: 34.1 ± 6.9 years; Control: 33.8 ± 6.8 years	PCOS: 36.0 ± 6.6 kg/m^2^; Control: 37.4 ± 5.6 kg/m^2^	PCOS: 0.86 ± 0.08; Control: 0.84 ± 0.06	PCOS: 4.8 ± 0.3 μmol/L; Control: 3.4 ± 0.4 μmol/L^*^	HDL-C, LDL-C, TG, nonHDL-C
Ni et al. (38)	China	Retrospective	578 vs. 281	PCOS: 27.3 ± 3.7 years; Control: 28.3 ± 3.7 years	PCOS: 22.1 ± 3.7 kg/m^2^; Control: 22.2 ± 2.2 kg/m^2^	-	PCOS: 5.4 ± 2.5 μmol/L; Control: 4.4 ± 1.9 μmol/L^*^	HDL-C, LDL-C, TG, SBP, DBP
Oral et al. (39)	Turkey	Prospective	48 vs. 43	PCOS: 23.9 ± 3.3 years; Control: 24.2 ± 3.9 years	PCOS: 24.1 ± 2.9 kg/m^2^; Control: 24.0 ± 1.9 kg/m^2^	-	PCOS: 256.3 ± 59.5 μg/dL; Control: 246.5 ± 59.5 μg/dl^*^	HDL-C, LDL-C, TG, TC, nonHDL-C
Orio et al. (40)	Italy	Prospective	30 vs. 30	PCOS: 22.2 ± 2.5 years; Control: 22.6 ± 2.3 years	PCOS: 22.4 ± 2.1 kg/m^2^; Control: 22.1 ± 1.8 kg/m^2^	PCOS: 0.77 ± 0.4; Control: 0.72 ± 0.3	PCOS: 4535 ± 527 μmol/L; Control: 2988 ± 311 μmol/L^*^	HDL-C, LDL-C, TG, SBP, DBP, nonHDL-C
Philbois et al. (41)	Brazil	Retrospective	60 vs. 30	PCOS without obese: 28.5 ± 5.2 years; PCOS without obese: 30.2 ± 5.3 years; Control: 31.2 ± 6.6 years	PCOS without obese: 22.95 ± 1.6 kg/m^2^; PCOS with obese: 33.9 ± 2.4 kg/m^2^; Control: 23.5 ± 3 kg/m2	-	-	SBP, DBP
Rizzo et al. (42)	Italy	Prospective	350 vs. 90	PCOS: 24 ± 5 years; Control: 24 ± 3 years	PCOS: 27 ± 7 kg/m^2^; Control: 27 ± 4 kg/m^2^	-	-	HDL-C, LDL-C, TG, nonHDL-C
Sasaki et al. (43)	Japan	Prospective	54 vs. 24	PCOS: 30.2 ± 3.9 years; Control: 31.5 ± 4.4 years	PCOS: 24.3 ± 5.7 kg/m^2^; Control: 22.2 ± 3.4 kg/m^2^	-	-	HDL-C, LDL-C, TG, SBP, DBP, nonHDL-C
Shafiee et al. (44)	UK	Cross-section	34 vs. 34	PCOS: 31.8 ± 5.97 years; Control: 43.68 ± 13.12 years	PCOS: 29.28 ± 2.91 kg/m^2^; Control: 28.58 ± 2.62 kg/m^2^	PCOS: 0.88 ± 0.03; Control: 0.85 ± 0.02	-	HDL-C, LDL-C, TG, SBP, DBP, nonHDL-C
Shroff et al. (45)	USA	Prospective	24 vs. 24	PCOS: 32 ± 6.5 years; Control: 36 ± 7.2 years	PCOS: 36 ± 5.4 kg/m^2^; Control: 35 ± 3.3 kg/m^2^	PCOS: 0.85 ± 0.1; Control: 0.82 ± 0.1	-	HDL-C, LDL-C, TG, nonHDL-C
Soares et al. (46)	Brazil	Cross-section	40 vs. 50	PCOS: 24.5 ± 3.8 years; Control: 24.5 ± 5.1 years	PCOS: 22.7 ± 3.3 kg/m^2^; Control: 23.1 ± 3.2 kg/m^2^	-	-	HDL-C, LDL-C, TG, SBP, DBP, nonHDL-C
Tarkun et al. (47)	Turkey	Prospective	37 vs. 25	PCOS: 23.45 ± 4.3 years; Control: 24.4 ± 4.07 years	PCOS: 23.85 ± 3.26 kg/m^2^; Control: 22.9 ± 2.97 kg/m^2^	-	PCOS: 4.08 ± 2.3 ng/ml; Control: 2.89 ± 1.1 ng/ml	HDL-C, LDL-C, TG, nonHDL-C
Tíras et al. (48)	Turkey	Prospective	35 vs. 35	PCOS: 24.5 ± 6.0 years; Control: 23.6 ± 3.9 years	PCOS: 22.9 ± 4.2 kg/m^2^; Control: 22.0 ± 1.8 kg/m^2^	-	PCOS: 4.05 ± 3.14 ng/ml; Control: 2.48 ± 0.98 ng/ml	HDL-C, LDL-C, TG, nonHDL-C
Vryonidou et al. (49)	Greece	Prospective	75 vs. 55	PCOS: 23.9 ± 5.4 years; Control: 24.7 ± 5.3 years	PCOS: 27.3 ± 7.0 kg/m^2^; Control: 26.3 ± 7.7 kg/m^2^	PCOS: 0.79 ± 0.07; Control: 0.75 ± 0.04	PCOS: 8.12 ± 9.11 μmol/L; Control: 6.54 ± 8.59 μmol/L^*^	HDL-C, LDL-C, TG, TC, SBP, nonHDL-C
Yildiz et al. (50)	Turkey	Prospective	595 vs. 23	PCOS: 22.9 ± 4.4 years; Control: 24.8 ± 4.2 years	PCOS: 23.0 ± 2.4 kg/m^2^; Control: 22.1 ± 2.2 kg/m^2^	PCOS: 0.76 ± 0.02; Control: 0.71 ± 0.24	PCOS: 9.4 ± 3.1 nmol/L; Control: 5.9 ± 1.7 nmol/L	HDL-C, TG, nonHDL-C

* *Represented that these studies measured the level of dehydroepiandrosterone sulfate, others measured androstenedione level. WHR, Waist-to-hip ratio; BMI, Body mass index; LDL-C, low-density lipoprotein cholesterol; TG, triglyceride; HDL-C, low high-density lipoprotein cholesterol; SBP, Systolic blood pressure; DBP, Diastolic blood pressure*.

### Statistical Analysis

Continuous normally distributed data were summarized with means and standard deviations (SDs), and non-normally distributed variables were summarized with medians and interquartile ranges (IQRs). If the outcome was measured on the same scale, we used the weighted mean difference (MD) and 95% CI. Otherwise, standardized mean difference (SMD) and 95% CI were calculated. Random-effects models were used to calculate estimates. A *p*-value < 0.05 was considered statistically significant for all analyses except heterogeneity tests.

We assessed the possibility of publication bias by constructing a funnel plot of each trial's effect size against its standard error. We assessed funnel plot asymmetry using the Egger's regression test and defined significant publication bias as a *p*-value < 0.1. The trim-and-fill computation was used to estimate the effect of publication bias on the interpretation of results (51). Heterogeneity between studies was assessed using *I*^2^ tests (*I*^2^> 50% was considered substantial heterogeneity). Analyses were performed by the Stata statistical software version 14.0 (StataCorp, College Station, TX, USA).

## Results

### Study Design and Analysis Characteristics

**Figure 1:**
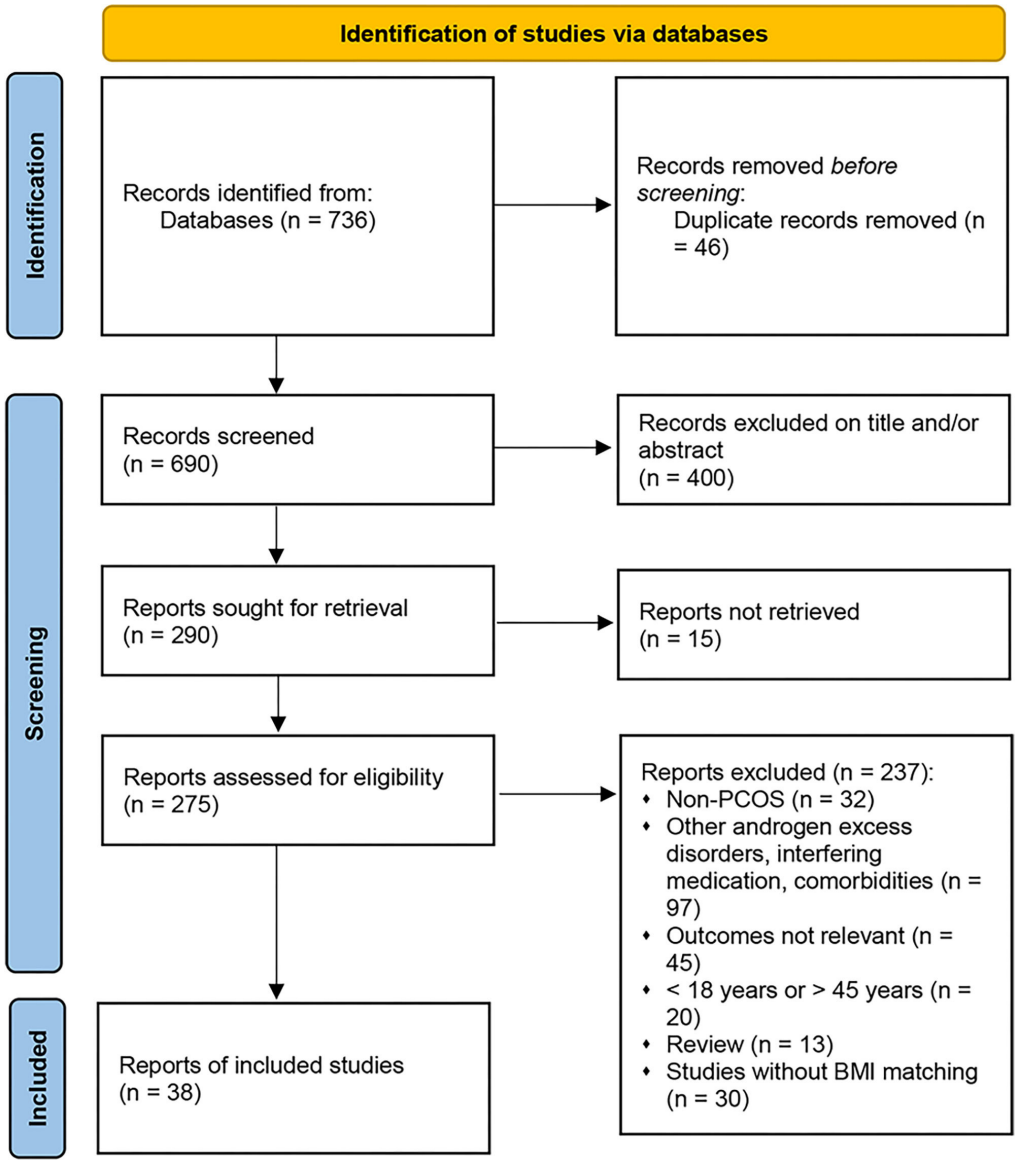
PRISMA flow chart of the study selection procedure.

The search yielded 736 potential reports, as outlined in the PRISMA flow diagram (Figure 1). After the removal of duplicates, 690 records remained. Initial screening of the titles and abstracts resulted in the exclusion of 400 reports, and 275 studies proceeded for detailed evaluation. After further examination, 13 cross-sectional studies and 25 cohort studies met the inclusion criteria and were included in the metaanalysis (13–16, 18–50, 52). The basic characteristics of each study are summarized in Table 2. A total of 38 studies and 6,078 subjects were included in the present metaanalysis which assessed HDL-C, TGs, nonHDL-C and LDL-C, SBP, DBP, and WHR according to BMI categories.

### Risk of Bias and Quality Assessment

The 38 studies included in our metaanalysis were reviewed with the NOS tool. One study scored seven out of nine points, and one study scored six points, both indicating high quality. Three studies scored four points or less. The details are provided in Table 3.

**Table 3:** Assessment of methodological quality (based on Newcastle-Ottawa Scale).

**References**	**Selection (max 4 stars)**	**Comparability (max 2 stars)**	**Exposure (max 3 stars)**	**Overall quality**
Adali et al. (14)	***	**	**	Good
Akram et al. (13)	**	**	**	Fair
Alexandraki et al. (15)	***	**	**	Good
Arikan et al. (16)	***	**	**	Good
Berneis et al. (52)	****	**	**	Good
Calzada et al. (18)	***	**	**	Good
Cascella et al. (18)	****	**	**	Good
Cetinakalp et al. (20)	**	**	**	Fair
Cheng et al. (21)	***	**	**	Good
Cussons et al. (22)	**	**	*	Fair
Diamanti-Kandarakis et al. (23)	**	**	*	Fair
El-Kannishy et al. (24)	****	**	**	Good
Erdogan et al. (25)	**	*	**	Fair
Erdogan (25]	****	**	**	Good
Glintborg et al. (27)	***	**	**	Good
González et al. (28)	***	**	**	Good
Kargili et al. (29)	**	**	**	Fair
Ketel et al. (30)	****	**	**	Good
Legro et al. (31)	***	*	**	Good
Liang et al. (32)	***	*	**	Good
Long et al. (33)	***	**	**	Good
Luque-Ramirez et al. (34)	***	**	**	Good
Macut et al. (35)	**	**	**	Fair
Moran et al. (37)	***	**	**	Good
Mayer et al. (36)	****	**	**	Good
Ni et al. (38)	**	*	**	Fair
Oral et al. (39)	**	*	**	Fair
Orio et al. (40)	***	**	**	Good
Philbois et al. (41)	**	**	**	Fair
Rizzo et al. (42)	**	**	**	Fair
Sasaki et al. (43)	***	**	**	Good
Shafiee et al. (44)	***	**	**	Good
Shroff et al. (45)	***	**	**	Good
Soares et al. (46)	**	**	**	Fair
Tarkun et al. (47)	**	**	**	Fair
Tíras et al. (48)	**	**	**	Fair
Vryonidou et al. (49)	****	**	**	Good
Yildiz et al. (50)	**	*	**	Fair

*SELECTION*

(1) Is the case definition adequate? (a) yes, with independent validation^*^, (b) yes, e.g., record linkage or based on self-reports, (c) no description.

(2) Representativeness of the cases: (a) consecutive or obviously representative series of cases^*^, (b) potential for selection biases or not stated.

(3) Selection of controls: (a) community controls^*^, (b) hospital controls, (c) no description.

(4) Definition of controls: (a) no history of disease (end-point)^*^, (b) no description of source.

*COMPARABILITY*

Comparability of cases and controls on basis of design or analysis: (a) study controls for ___ (most important factor)^*^, (b) study controls for any additional factor^*^ (could be modified to indicate specific control for a second factor).

*EXPOSURE*

(1) Ascertainment of exposure: (a) secure record (e.g., surgical record)^*^, (b) structured interview where blind to case/control status^*^, (c) interview not blinded to case/control status, (d) written self-report or medical record only, (e) no description.

(2) Same method of ascertainment for cases and controls: (a) yes^*^, (b) no.

(3) Non-response rate: (a) same rate for both groups^*^, (b) non-respondents describe, (c) rate different and no designation.

*OVERALL QUALITY*

*Good quality: 3 or 4 stars in selection domain AND 1 or 2 stars in comparability domain AND 2 or 3 stars in exposure domain*.

*Fair quality: 2 stars in selection domain AND 1 or 2 stars in comparability domain AND 2 or 3 stars in exposure domain*.

*Poor quality: 0 or 1 star in selection domain OR 0 star in comparability domain OR 0 or 1 star in exposure domain*.

### Lipid Profiles

**Figure 2:**
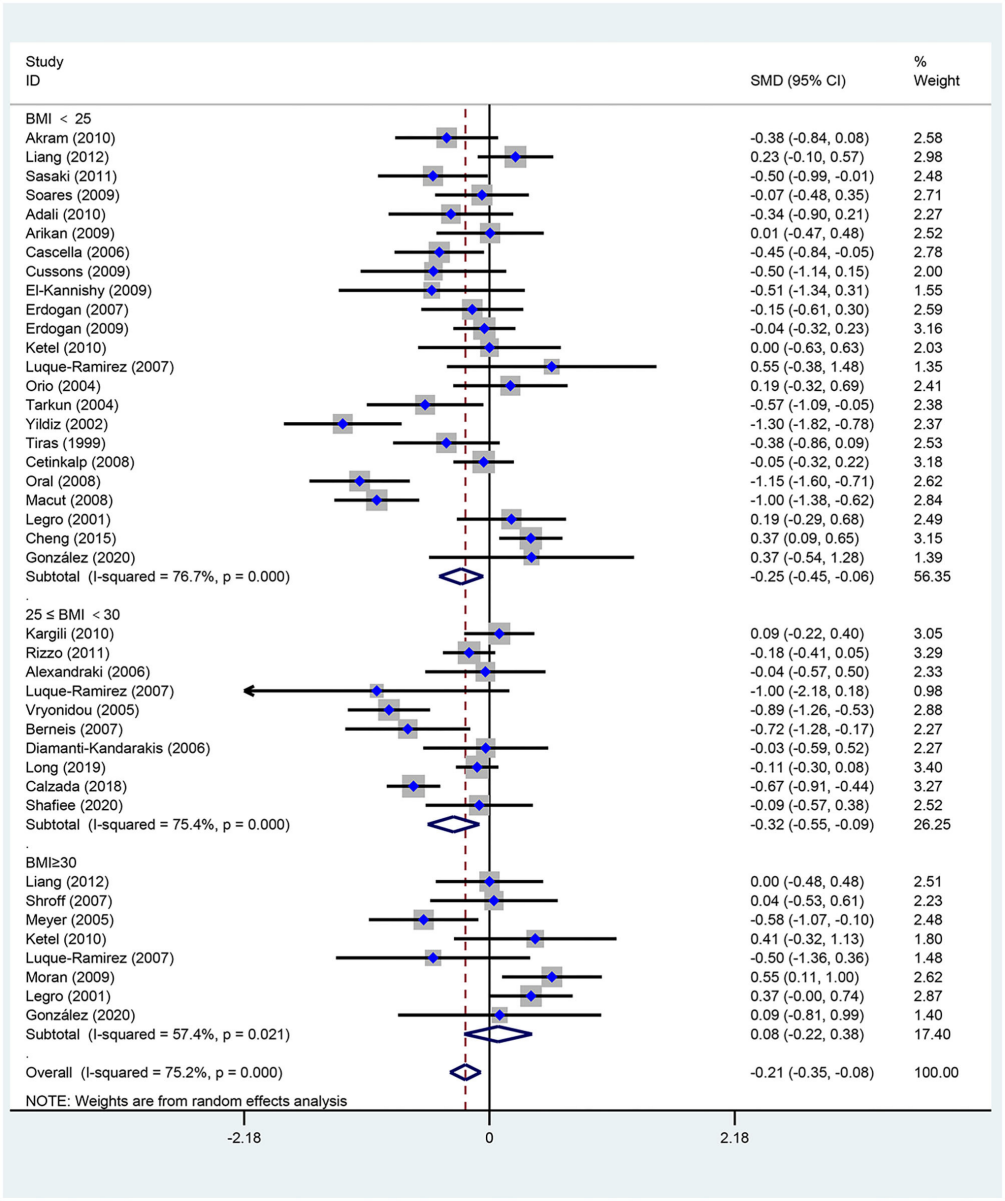
Forest plot for comparison of high-density lipoprotein-cholesterol in subjects with polycystic ovary syndrome (PCOS) vs. control subjects. Studies are classified by different body mass index (BMI) categories (BMI <25 kg/m^2^, BMI ≥ 30 kg/m^2^, and BMI 25–30 kg/m^2^).

**Figure 3:**
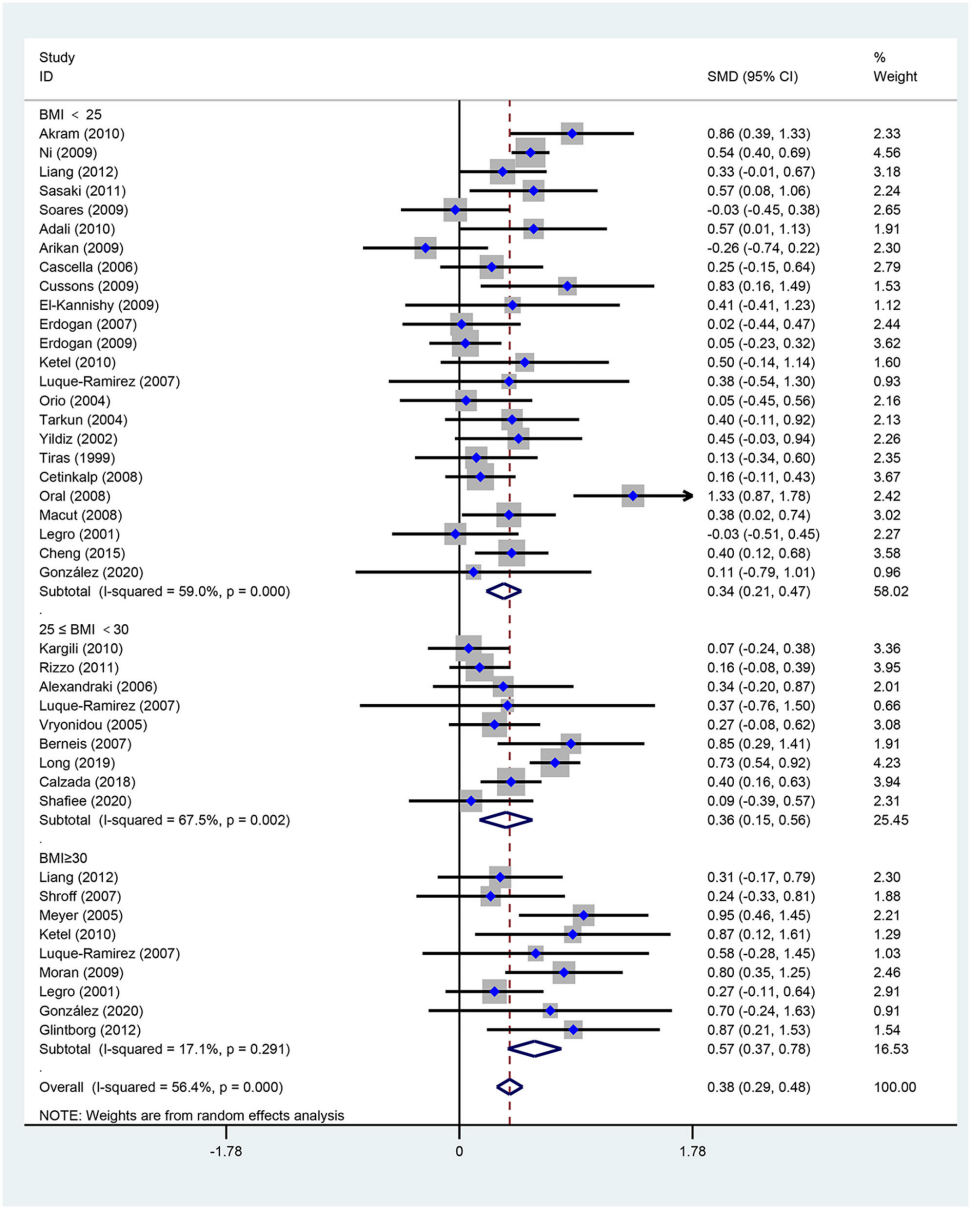
Forest plot for comparison of triglycerides in polycystic ovary syndrome vs. control subjects. Studies are classified by different body mass index (BMI) categories (BMI <25 kg/m^2^, BMI ≥ 30 kg/m^2^, and BMI 25–30 kg/m^2^).

**Figure 4:**
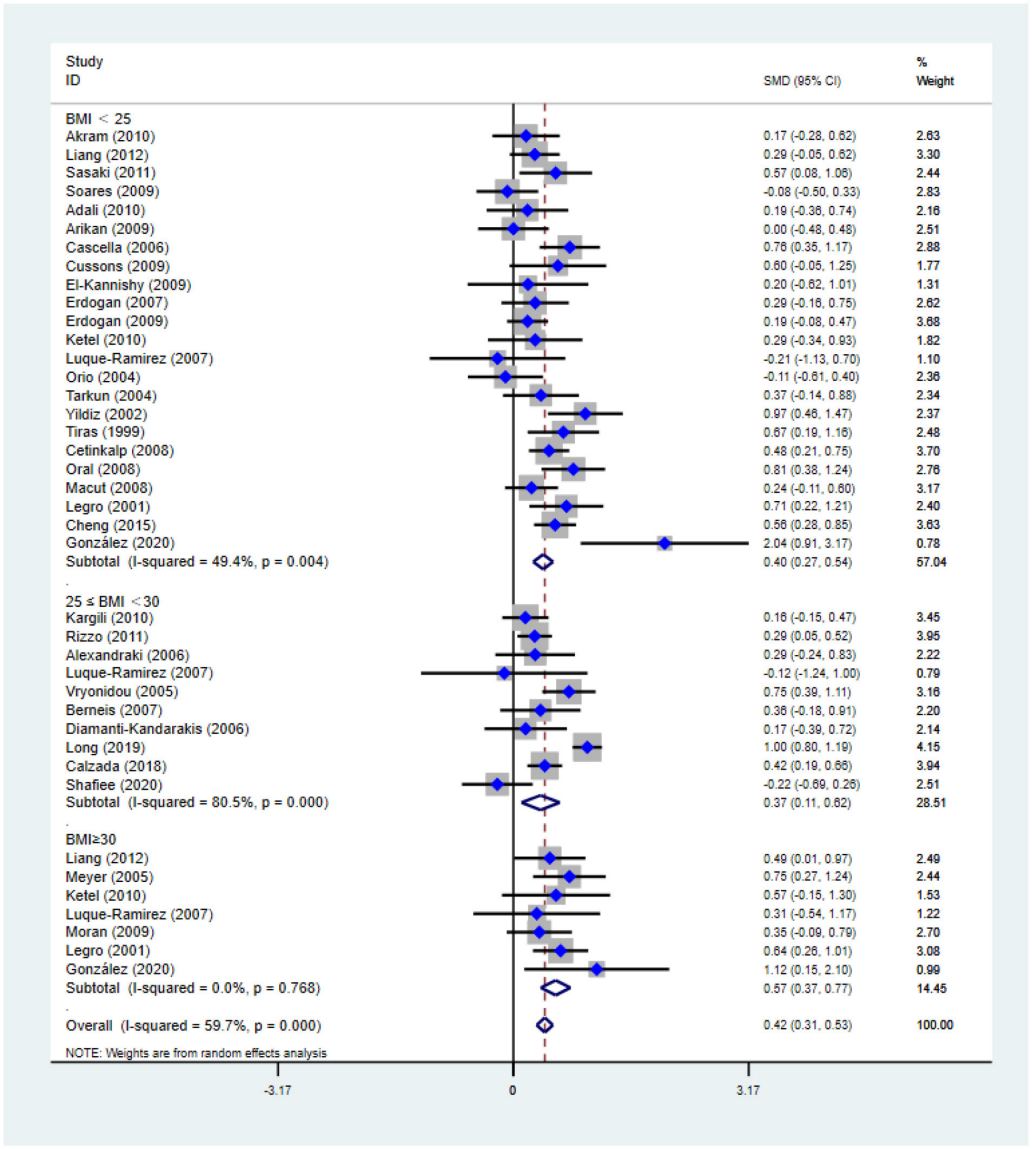
Forest plot for comparison of non-high-density lipoprotein-cholesterol in polycystic ovary syndrome vs. control subjects. Studies are classified by different body mass index (BMI) categories (BMI < 25 kg/m^2^, BMI ≥ 30 kg/m^2^, and BMI 25–30 kg/m^2^).

**Figure 5:**
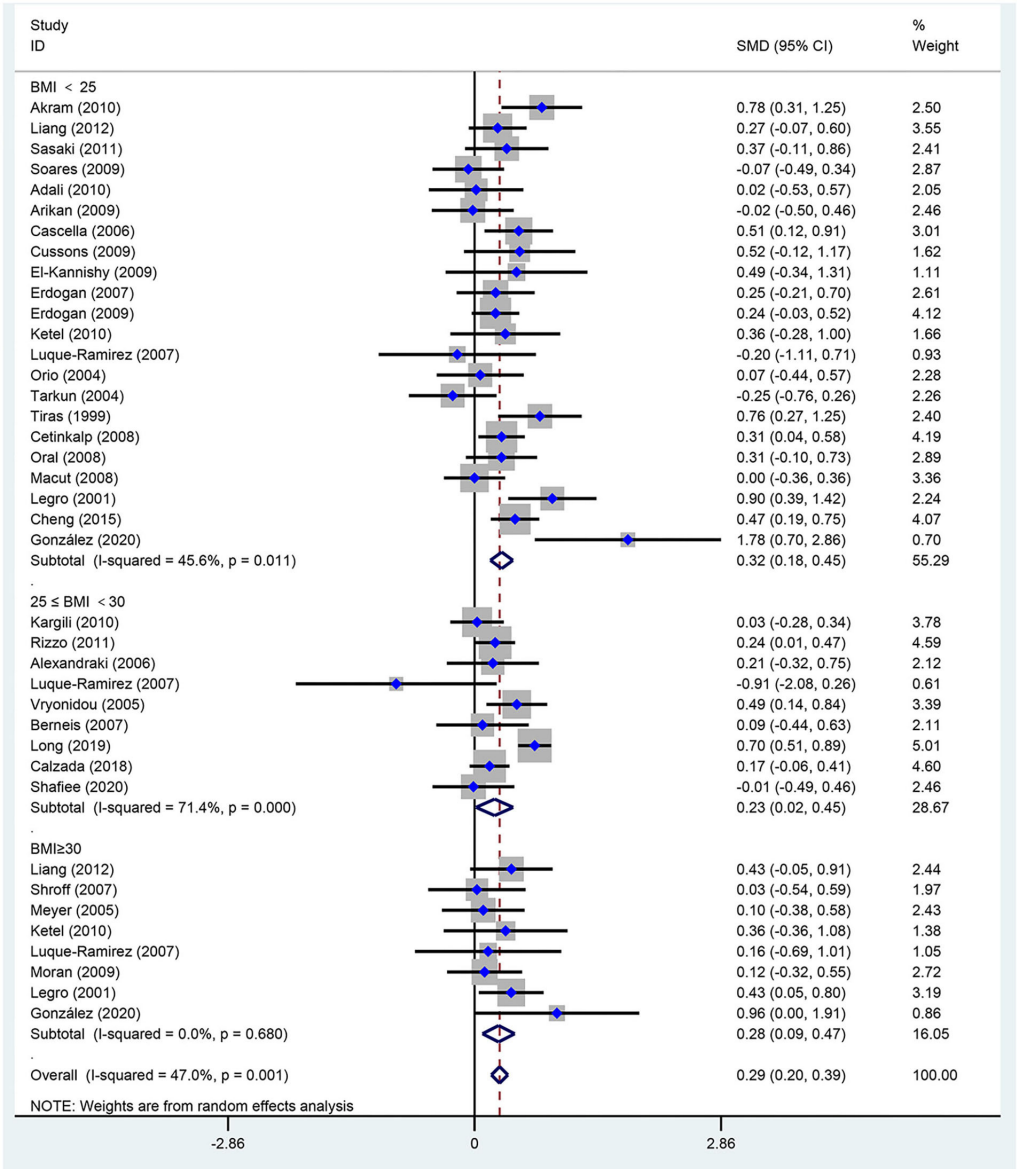
Forest plot for comparison of low-density lipoprotein-cholesterol in polycystic ovary syndrome vs. control subjects. Studies are classified by different body mass index (BMI) categories (BMI <25 kg/m^2^, BMI ≥ 30 kg/m^2^, and BMI 25–30 kg/m^2^).

Figures 2–5 are forest plots summarizing the comparisons of HDL-C, TG, nonHDL-C, and LDL-C, respectively.

As shown in Figure 2, women of reproductive age with PCOS had a significant decrease in HDL-C [SMD (95% CI): −0.21 (−0.35, −0.08), *p* = 0.002], with statistically significant between-study heterogeneity. The subgroup analysis showed that HDL-C significantly decreased in BMI <25 kg/m^2^ [SMD (95% CI): −0.25 (−0.45, −0.06), *p* = 0.012], and BMI 25–30 kg/m^2^ [MD (95% CI): −0.32 (−0.55, −0.09), *p* = 0.006]. However, HDL-C did not significantly differ at BMI ≥ 30 kg/m^2^ [MD (95% CI): 0.08 (−0.22, 0.38), *p* = 0.618]. There was no significant publication bias in this analysis (asymmetry test *P* = 0.680). Further sensitivity analysis comparing PCOS patients and controls showed a mean reduction in HDL-C in PCOS patients.

As illustrated in Figure 3, women of reproductive age with PCOS had a significant difference in TGs [SMD (95% CI): 0.38 (0.29, 0.48), *p* < 0.001], with significant between-study heterogeneity. The subgroup analysis of TGs revealed that TGs were increased in women of reproductive age with PCOS at BMI <25 kg/m^2^ [SMD (95% CI): 0.34 (0.21, 0.47), *p* < 0.001], BMI ≥ 30 kg/m^2^ [MD (95% CI): 0.57 (0.37, 0.78), *p* < 0.001], and BMI 25–30 kg/m^2^ [MD (95% CI): 0.36 (0.15, 0.56), *p* = 0.001]. In this analysis, there was no publication bias (asymmetry test *P* = 0.882). In the sensitivity analysis comparing PCOS patients and controls, we observed an increase in TGs in women with PCOS.

As shown in Figure 4, non-HDL-C [SMD (95% CI): 0.42 (0.31, 0.53), *p* < 0.001] increased in reproductive-aged women with PCOS, with significant between-study heterogeneity. The subgroup analysis showed that non-HDL-C increased in women with PCOS at the three BMI levels, including BMI < 25 kg/m^2^ [SMD (95% CI): 0.40 (0.27, 0.54), *p* < 0.001], BMI ≥ 30 kg/m^2^ [MD (95% CI): 0.57 (0.37, 0.77), *p* < 0.001], and BMI 25–30 kg/m^2^ [MD (95% CI): 0.37 (0.11, 0.62), *p* = 0.005]. In this analysis, no publication bias was evident (asymmetry test *P* = 0.291). In the sensitivity analysis, women of reproductive age with PCOS had an increase in nonHDL-C compared with that of controls.

As seen in Figure 5, women of reproductive age with PCOS had an increased level of LDL-C [SMD (95% CI): 0.29 (0.20, 0.39), *p* < 0.001], with no significant between-study heterogeneity. In addition, LDL-C increased in women with PCOS at BMI <25 kg/m^2^ [SMD (95% CI): 0.32 (0.18, 0.45), *p* < 0.001], BMI ≥ 30 kg/m^2^ [MD (95% CI): 0.28 (0.09, 0.47), *p* = 0.003] and BMI 25–30 kg/m^2^ [MD (95% CI): 0.23 (0.02, 0.45), *p* = 0.034]. There was no publication bias (asymmetry test *P* = 0.414). In the sensitivity analysis, women of reproductive age with PCOS showed an increase in LDL-C compared with that of controls.

### Blood Pressure

**Figure 6:**
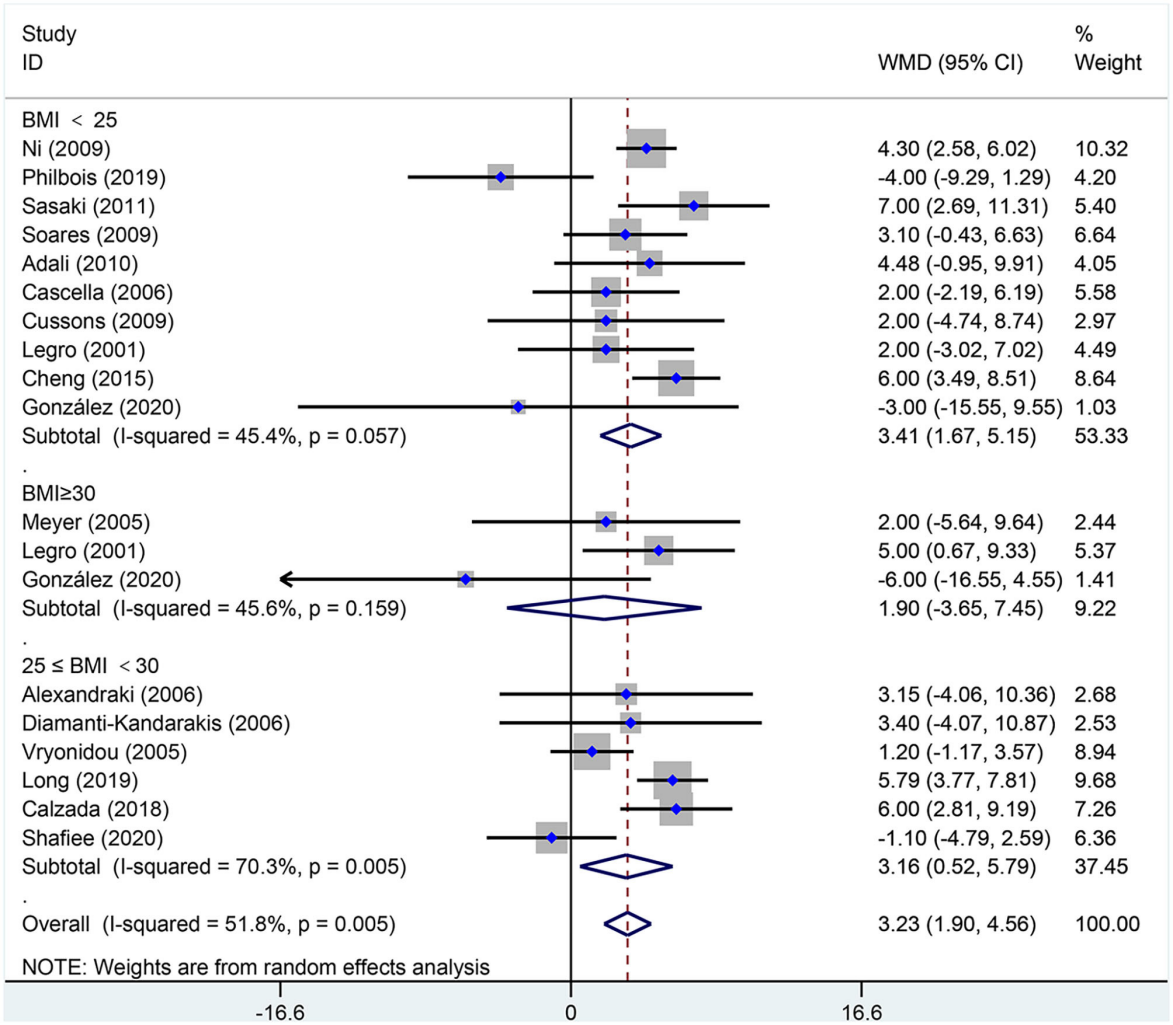
Forest plot for comparison of systolic blood pressure in polycystic ovary syndrome vs. control subjects. Studies are classified by different body mass index (BMI) categories (BMI <25 kg/m^2^, BMI ≥ 30 kg/m^2^, and BMI 25–30 kg/m^2^).

**Figure 7:**
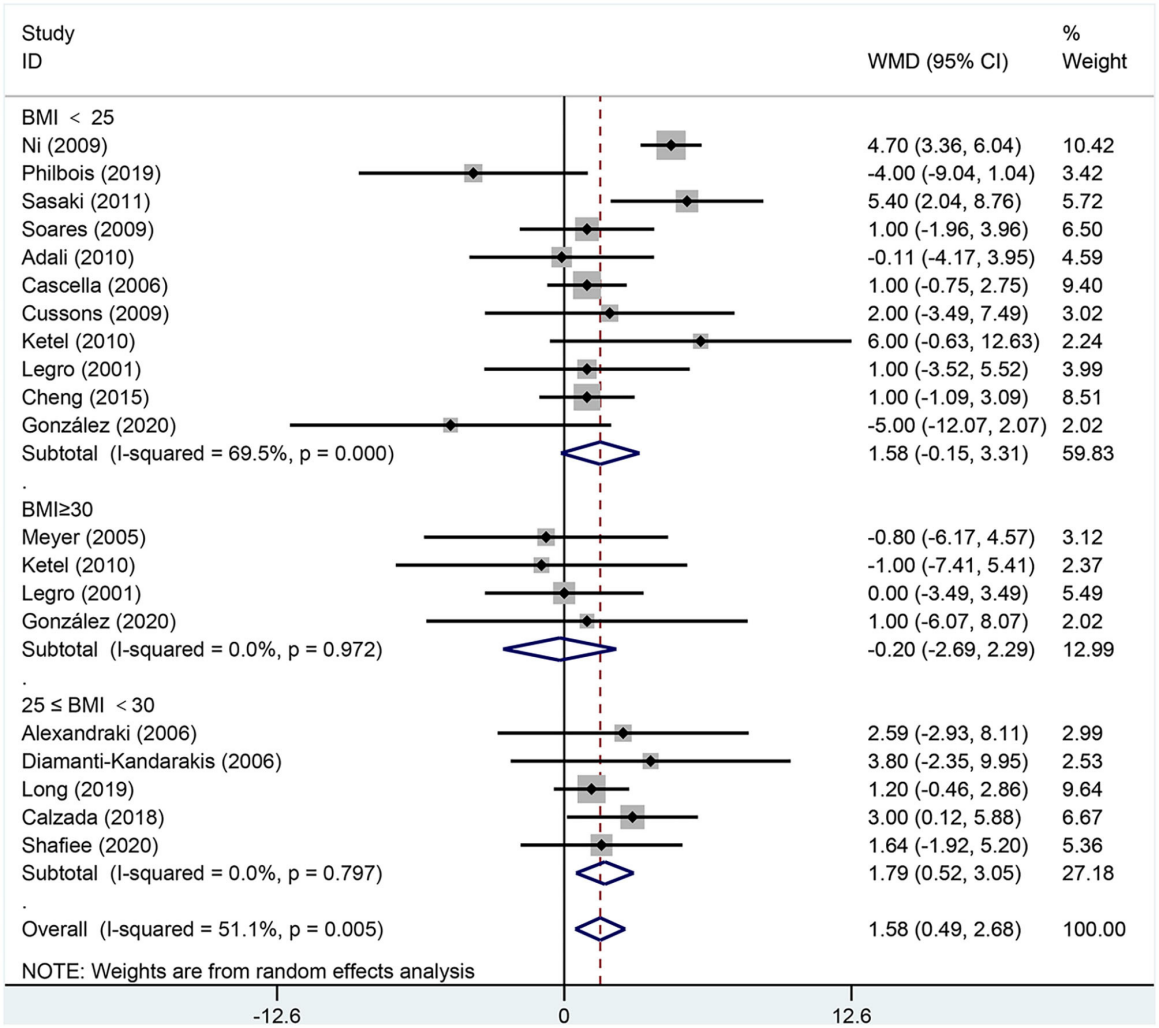
Forest plot for comparison of diastolic blood pressure in polycystic ovary syndrome vs. control subjects. Studies are classified by different body mass index (BMI) categories (BMI <25 kg/m^2^, BMI ≥ 30 kg/m^2^, and BMI 25–30 kg/m^2^).

Figures 6, 7 are forest plots summarizing comparisons of SBP and DBP, respectively.

As shown in Figure 6, SBP [MD (95% CI): 3.23 mmHg (1.90, 4.56), *p* < 0.001] increased in women of reproductive age with PCOS. In the subgroup analysis of SBP, SBP increased in women with PCOS at BMI <25 kg/m^2^ [MD (95% CI): 3.41 mmHg (1.67, 5.15), *p* < 0.001] and BMI 25–30 kg/m^2^ [MD (95% CI): 3.16 mmHg (0.52, 5.79), *p* = 0.019]; however, women with PCOS at BMI ≥ 30 kg/m^2^ [MD (95% CI): 1.90 mmHg (−3.65, 7.45), *p* = 0.502] did not differ in SBP. In this analysis, the Egger's test detected publication bias (asymmetry test *p* = 0.062). However, further analysis with a trim-and-fill test indicated that this publication bias did not impact the estimates (i.e., no trimming was done because the data were unchanged). Further sensitivity analysis comparing PCOS patients and controls showed an increase in SBP in PCOS patients.

As shown in Figure 7, DBP [MD (95% CI): 1.58 mmHg (0.49, 2.68), *p* = 0.005] increased in women of reproductive age with PCOS. In the subgroup analysis of DBP in women with PCOS, BMI 25–30 kg/m^2^ [MD (95% CI): 1.79 mmHg (0.52, 3.05), *p* = 0.006] showed positive results, while DBP did not increase at BMI <25 kg/m^2^ [MD (95% CI): 1.58 mmHg (−0.15, 3.31), *p* = 0.073] or BMI ≥ 30 kg/m^2^ [MD (95% CI): −0.20 mmHg (−2.69, 2.29), *p* = 0.876]. In this analysis, there was publication bias as detected by the Egger's test (asymmetry test *P* = 0.098). However, further analysis with a trim-and-fill test indicated that this publication bias did not impact the estimates.

### Waist-to-Hip Ratio

**Figure 8:**
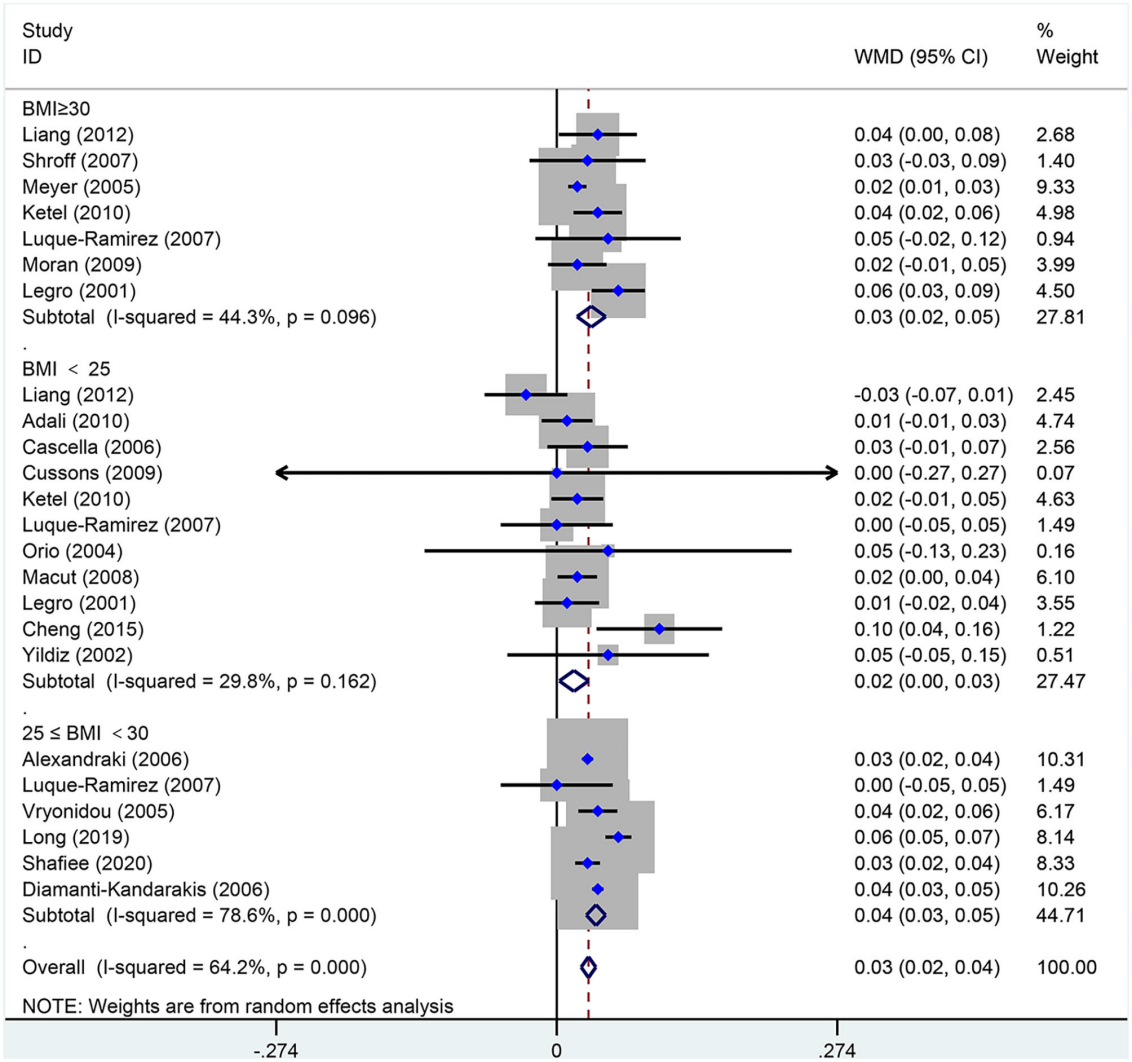
Forest plot for comparison of waist-to-hip ratio in polycystic ovary syndrome vs. control subjects. Studies are classified by different body mass index (BMI) categories (BMI <25 kg/m^2^, BMI ≥ 30 kg/m^2^, and BMI 25 <30 kg/m^2^).

As shown in Figure 8, WHR [MD (95% CI): 0.03 (0.02, 0.04), *p* < 0.001] increased in women of reproductive age with PCOS. In the subgroup analysis of WHR in women with PCOS, BMI <25 kg/m^2^ [MD (95% CI): 0.02 (0.00, 0.03), *p* = 0.022], BMI 25–30 kg/m^2^ [MD (95% CI): 0.04 (0.03, 0.05), *p* < 0.001] and BMI ≥ 30 kg/m^2^ [MD (95% CI): 0.03 (0.02, 0.05), *p* < 0.001] showed positive results. In this analysis, there was no publication bias on Egger's test (asymmetry test *P* = 0.547).

## Discussion

This systematic review of 38 observational studies reports the correlation between cardiovascular risk and BMI for women of reproductive age with PCOS. To the best of our knowledge, this is the first time that BMI categories were evaluated as cardiovascular risk factors in women of reproductive age with PCOS. Dyslipidemia and high baseline blood pressure are common in women of reproductive age with PCOS, and earlier focus had been on alterations in TGs and HDL-C. This metaanalysis showed that LDL-C and nonHDL-C increased across all BMI categories for women with PCOS. LDL-C and nonHDL-C, which are critical targets for preventing atherosclerosis, may be more important for the prevention of cardiovascular risk. In addition, blood pressure should also be considered.

In the present metaanalysis, increased baseline blood pressure was seen in women of reproductive age with PCOS. SBP and DBP were considerably higher in PCOS subjects whose BMI was between 25 and 30 kg/m^2^. Moreover, SBP alone increased in PCOS subjects whose the BMI was <25 kg/m^2^. However, there was no difference in SBP and DBP in PCOS subjects whose BMI was more than 30 kg/m^2^. One cross-sectional Brazilian study on 233 women with PCOS and 70 controls found a higher prevalence of hypertension among the PCOS group (53). The prevalence of hypertension was 65% among women with PCOS (mean age: 25-26.5 years) and 41% among control women without PCOS (mean age: 29 years) in that study (53). Zimmermann et al. reported that BMI did not reveal an association between women of reproductive age with PCOS and hypertension (54). A study from Australia reported that hypertension in women of reproductive age with PCOS was not associated with BMI, further indicating that cardiometabolic abnormalities may be independent of weight in women with PCOS (55). Because of the small sample size, we did not evaluate the prevalence of hypertension. However, the present metaanalysis found that baseline blood pressure was higher in women of reproductive age with PCOS than in age- and BMI-matched controls. Schmidt et al. showed that high blood pressure and hypertriglyceridemia were the only cardiovascular risk factors that persisted in postmenopausal women with PCOS. The main factors for increased blood pressure in women with PCOS, include endothelial dysfunction and decreased vascular compliance (56). The increased risk of hypertension can be explained by insulin resistance and hyperinsulinemia, which result in hypertrophy of the vascular muscle wall and reduce vascular compliance by interfering with endothelium-dependent vasodilatation mechanisms (57). Hyperinsulinemia promotes endothelin-1 (ET-1) production and influences its hypertrophic effect on vascular endothelial and smooth muscle cells (56). In addition, ET-1, which regulates endothelial function, is commonly chronically increased in women with PCOS.

The present meta-analysis showed dyslipidemia in women of reproductive age with PCOS, as increased LDL-C, nonHDL-C and TGs were found across all BMI categories. These findings are consistent with those of previous studies (58, 59). Women with PCOS are known to have increased levels of atherogenic apoB-containing particles, with a predominance of LDL particles that are smaller and have higher density (17). Small, dense LDL particles are the most atherogenic LDL particles and are strongly associated with cardiovascular risk due to their enhanced susceptibility to oxidation, reduced affinity to LDL receptor and greater arterial entry and retention (60). Women with PCOS younger than 40 appear to have dyslipidemia (59). Therefore, PCOS *per se* increases lipid levels, although the absolute value of the resultant lipid levels and the related cardiovascular risk may differ among individual patients. This lipid pattern assessment is an important finding for decreasing cardiovascular risk in women of reproductive age with PCOS women across all BMI categories. Subgroup analyses from a previous metaanalysis showed that higher LDL-C was found in women with higher BMI categories (61). However, our finding that women of reproductive age with PCOS showed that increased LDL-C was seen across all BMI categories. Because the measurement of LDL-C can be influenced by increased TGs in cardiovascular risk assessment (62), an appropriate way to estimate the amount of apo B-containing lipoproteins is to determine nonHDL-C (63). In addition, nonHDL-C has a comparable prognostic relevance to LDL-C (64, 65). We found that nonHDL-C increased in all BMI categories. It is important to evaluate nonHDL-C levels in women of reproductive age with PCOS. Increase in the levels of nonHDL-C, particularly at young ages, predicts long-term cardiovascular risks. This suggests that obesity (or being overweight) is not the largest cardiovascular risk factor in women of reproductive age with PCOS. Because the atherosclerotic process starts early in life, this confirms the need to assess and eventually treat altered lipid profiles in young women with PCOS (66).

Although obesity is an important confound in the relationship between PCOS diagnosis and cardiovascular risk, non-obese subjects also have a high dyslipidemia risk (56, 67). In addition to the reproductive and psychological symptoms of PCOS, the metabolic aberrations of PCOS worsen with obesity (68). Notably, non-obese women with PCOS are also at an increased risk of similar cardiometabolic aberrations. Women with PCOS have enlarged adipocytes in subcutaneous adipose tissue (SAT), lower circulating levels of adiponectin and increased abdominal adiposity independent of BMI (69–71). Although it was long postulated that SAT was less relevant than visceral adipose tissue (VAT) for metabolic dysfunction, SAT has recently been linked with metabolic alterations as well (72). Enlarged adipocytes in the adipose tissue of women with PCOS are associated with decreased adiponectin production and increased insulin resistance (IR), which indicates alterations in the function and morphology of adipose tissue as a PCOS process (70). In addition, women with PCOS had increased accumulations of adipose tissue in abdominal depots, predisposing them to the development of metabolic diseases, such as IR and diabetes, or to a higher risk for metabolic sequelae (73, 74). Adipose tissue dysfunction further correlates with an adverse metabolic profile. The close relationship between PCOS and cardiovascular risk is independent of BMI but may be due to SAT and VAT. In this metaanalysis, we found WHR increased in all BMI categories. Therefore, it is necessary to screen lipid levels, especially nonHDL, for women of reproductive age with PCOS of all BMI categories. We have shown that PCOS is associated with an increased cardiovascular risk. Although increased BMI was not the sole cause of the increased risk for cardiovascular events, the role of BMI should be examined in more detail in future studies. Dyslipidemia is common in women of reproductive age with PCOS. Furthermore, nonHDL-C plays an important role in cardiovascular risk in women with PCOS.

Another feature of PCOS, hyperandrogenism, results in excessive androgen. Excessive androgen can independently aggravate the development of cardiovascular risk in women with PCOS (75). Excessive androgen increases carotid intima–media thickness and calcification in the coronary and aortic arteries in women with PCOS, which may reflect dyslipidemia induced by androgen excess (34, 76). Reports have shown that high blood pressure is positively correlated with androgen excess in women (75). Moreover, lipid-induced inflammation may stimulate androgen excess in women with PCOS (28). Androgen can induce inflammation and oxidative stress in the vascular endothelium and increase renal reabsorption of sodium and water, which indirectly decreases circulating HDL-C levels and LDL-C clearance (77). Thus, excess androgen plays a role in the development of hypertension and the progression of atherosclerosis (78).

Several limitations of this metaanalysis should be noted. First, PCOS is a heterogeneous disease, and not all phenotypes were examined in this metaanalysis. Second, there was significant clinical and statistical heterogeneity in the pooled analysis. This could be due to confounding effects related to factors such as age, BMI, study quality, ethnicity, PCOS phenotypes, or other clinical features. In addition, we used country regions as a proxy for ethnicity in this metaanalysis due to a lack of participant ethnic compositions in most studies. Further studies should include the ethnicity of participants and different phenotypes of PCOS to provide a better understanding of the differential effects of these factors on PCOS or metabolic syndromes.

In conclusion, high baseline blood pressure and dyslipidemia are common in women of reproductive age with PCOS and are characterized by high SBP and DBP, low HDL-C, and increased TGs, nonHDL-C and LDL-C. These lipid parameters and BPs are worse in women of reproductive age with PCOS than in controls, regardless of BMI. Lipid profiles (i.e., TGs, LDL-C and nonHDL-C) should receive increased attention in all women of reproductive age with PCOS.

## Data Availability Statement

The original contributions presented in the study are included in the article/supplementary material, further inquiries can be directed to the corresponding author/s.

## Author Contributions

CZ and JY designed this work. CZ and XL are responsible for the acquisition and analysis of data and also drafted the work. WW, RS, and MQ are responsible for acquiring data as well. All authors contributed to the article and approved the submitted version.

## Funding

This work was supported by the grants of National Natural Science Foundation of China (Nos. 81960086 and 81670385), Innovation Ability Promotion Project of Colleges and Universities in Gansu Province, China (No. 2019B-017), and Cuiying Science and Technology Innovation Project of Lanzhou University Second Hospital, China (No. CY2018-QN05).

## Conflict of Interest

The authors declare that the research was conducted in the absence of any commercial or financial relationships that could be construed as a potential conflict of interest.

## Publisher's Note

All claims expressed in this article are solely those of the authors and do not necessarily represent those of their affiliated organizations, or those of the publisher, the editors and the reviewers. Any product that may be evaluated in this article, or claim that may be made by its manufacturer, is not guaranteed or endorsed by the publisher.
