# Corrigendum: Cardiovascular risk according to body mass index in reproductive-aged women with polycystic ovary syndrome: a systematic review and meta-analysis

**DOI:** 10.3389/fcvm.2023.1186990

**Published:** 2023-06-19

**Authors:** Chenchen Zhuang, Xufei Luo, Wenjuan Wang, Runmin Sun, Miaomiao Qi, Jing Yu

**Affiliations:** ^1^ Hypertension Center, Lanzhou University Second Hospital, Lanzhou, China; ^2^ School of Public Health, Lanzhou University, Lanzhou, China

**Keywords:** polycystic ovary syndrome, cardiovascular risk, reproductive-age, meta-analysis, body mass index

A Corrigendum on Cardiovascular risk according to body mass index in women of reproductive age with polycystic ovary syndrome: a systematic review and meta-analysis By Zhuang C, Luo X, Wang W, Sun R, Qi M, and Yu J. (2022) Front. Cardiovasc Med. 9:822079. doi: 10.3389/fcvm.2022.822079

**Error in Figure/Table**

In the published article, there was an error in [**Table 1** **Search strategies**] as published. [We had searched PubMed for the first set of results in Table 1 and not PubMed-MEDLINE]**.** The corrected caption in [**Table 1** **Search strategies**] is **[PubMed] which appears below.

**PubMed**

In the published article, there was an error in [Table 2 Characteristics of included studies in the meta-analysis]. The corrected [Table 2 Characteristics of included studies in the meta-analysis] and its caption **[Table 2 Characteristics of included studies in the meta-analysis] appear below.

**Table 2:** Characteristics of included studies in the meta-analysis.

Author/Year	Place of study	Type of study	PCOS vs. control (n)	Participants age	BMI	WHR	Androgen level	Outcomes
Akram (2010)	Pakistan	Retrospective	50 vs. 30	20–39 years	PCOS: 23.6 ± 0.50 kg/m^2^; Control: 23.5 ± 0.71 kg/m^2^	–	–	HDL-C, LDL-C, TG, nonHDL-C
Adali (2010)	Turkey	Prospective	26 vs. 25	PCOS: 24.73 ± 2.91 years; Control: 25.04 ± 2.26 years	PCOS: 24.40 ± 4.23 kg/m^2^; Control: 23.90 ± 3.95 kg/m^2^	PCOS: 0.74 ± 0.05; Control: 0.73 ± 0.04	–	HDL-C, LDL-C, TG, SBP, DBP, nonHDL-C
Alexandraki (2006)	Greece	Cross-section	27 vs. 27	PCOS: 25.41 ± 0.80 years; Control: 27.33 ± 0.83 years	PCOS: 27.42 ± 1.12 kg/m^2^; Control: 25.05 ± 1.19 kg/m^2^	PCOS: 0.78 ± 0.01; Control: 0.75 ± 0.01	PCOS: 10.85 ± 0.76 nmol/L; Control: 5.37 ± 0.38 nmol/L	HDL-C, LDL-C, TG, nonHDL-C, SBP, DBP
Arikan (2007)	Turkey	Prospective	39 vs. 30	PCOS: 22.82 ± 5.53 years; Control: 24.64 ± 4.22 years	PCOS: 21.48 ± 6.50 kg/m^2^; Control: 20.90 ± 6.04 kg/m^2^	–	PCOS: 2.98 ± 1.31 ng/ml; Control: 1.37 ± 0.89 ng/ml	HDL-C, LDL-C, TG, nonHDL-C
Berneis (2009)	Italy	Cross-section	30 vs. 24	PCOS: 25.1 ± 4.2 years; Control: 25.5 ± 3 years	PCOS: 28.4 ± 5.8 kg/m^2^; Control: 28 ± 4.4 kg/m^2^	–	–	HDL-C, LDL-C, TG, nonHDL-C
Cascella (2006)	Italy	Prospective	50 vs. 50	PCOS: 21.9 ± 2.7 years; Control: 22.2 ± 2.8 years	PCOS: 24.6 ± 2.5 kg/m^2^; Control: 24.4 ± 2.8 kg/m^2^	PCOS: 0.86 ± 0.1; Control: 0.83 ± 0.1	PCOS: 5.1 ± 0.7 nmol/L; Control: 1.4 ± 0.6 nmol/L	HDL-C, LDL-C, TG, SBP, DBP, nonHDL-C
Calzada (2018)	Spain	Retrospective	125 vs. 169	PCOS: 28.0 ± 5.0 years; Control: 30.0 ± 6.0 years	PCOS: 25.7 ± 7.1 kg/m^2^; Control: 22.3 ± 3.1 kg/m^2^	–	PCOS: 2.73 ± 1.35 ng/ml; Control: 2.17 ± 1.05 ng/ml	HDL-C, LDL-C, TG, SBP, DBP, nonHDL-C
Cetinkalp (2009)	Turkey	Prospective	129 vs. 91	PCOS: 24.58 ± 4.61 years; Control: 25.48 ± 3.38 years	PCOS: 24.47 ± 4.64 kg/m^2^; Control: 24.20 ± 3.31 kg/m^2^	–	–	HDL-C, LDL-C, TG, nonHDL-C
Cheng (2015)	China	Prospective	103 vs. 96	PCOS: 26 ± 4 years; Control: 26 ± 2 years	PCOS: 24.2 ± 5.3 kg/m^2^ Control: 20.5 ± 2.7 kg/m^2^	PCOS: 0.9 ± 0.3; Control: 0.8 ± 0.1	–	HDL-C, LDL-C, TG, SBP, DBP, nonHDL-C
Cussons (2009)	Australia	Cross-section	19 vs. 19	PCOS: 30.4 ± 5.54 years; Control: 34.44 ± 7.8 years	PCOS: 24.1 ± 2.9 kg/m^2^ Control: 22.9 ± 3.2 kg/m^2^	PCOS: 0.8 ± 0.1; Control: 0.8 ± 0.6	PCOS: 10.89 ± 3.99 nmol/L; Control: 8.54 ± 2.41 nmol/L	HDL-C, LDL-C, TG, SBP, DBP, nonHDL-C
Diamanti-Kandarakis (2006)	Greece	Prospective	25 vs. 25	PCOS: 25.64 ± 0.86 years; Control: 27.52 ± 1.02 years	PCOS: 29.08 ± 1.43 kg/m^2^; Control: 26.22 ± 1.16 kg/m^2^	PCOS: 0.79 ± 0.01; Control: 0.75 ± 0.01	–	HDL-C, SBP, DBP, nonHDLC
El-Kannishy (2010)	Egypt	Cross-section	14 vs. 10	PCOS: 25.2 ± 3.6 years; Control: 24.4 ± 4.07 years	PCOS: 22.8 ± 2.1 kg/m^2^ Control: 21.9 ± 2.97 kg/m^2^	–	–	HDL-C, LDL-C, TG, nonHDL-C
Erdogan (2007)	USA	Retrospective	68 vs. 26	PCOS: 24.27 ± 5.44 years; Control: 26.41 ± 5.65 years	PCOS: 24.41 ± 5.43 kg/m^2^ Control: 23.35 ± 5.04 kg/m^2^	–	–	HDL-C, LDL-C, TG, nonHDL-C
Erdogan (2009)	USA	Retrospective	88 vs. 119	PCOS: 24.07 ± 1.32 years; Control: 25.01 ± 2.05 years	PCOS: 24.38 ± 4.13 kg/m^2^ Control: 23.47 ± 4.12 kg/m^2^	–	–	HDL-C, LDL-C, TG, nonHDL-C
Glintborg (2013)	Denmark	Prospective	30 vs. 14	PCOS: 32.3 ± 7.9 years; Control: 34.3 ± 12.4 years	PCOS: 33.5 ± 4.2 kg/m^2^; Control: 32.8 ± 7.1 kg/m^2^	–	–	TG
Gonzalez (2020)	USA	Cross-section	Lean: 10 vs. 10; Obese: 9 vs. 9	Lean: PCOS: 27 ± 1 years; Control: 29 ± 2 years; Obese: PCOS: 28 ± 2 years; Control: 32 ± 2 years	Lean: PCOS: 22.5 ± 0.6 kg/m^2^; Control: 22.0 ± 0.8 kg/m^2^; Obese: PCOS: 34.4 ± 0.9 kg/m^2^; Control: 34.1 ± 0.7 kg/m^2^	–	Lean: PCOS: 4.1 ± 0.4 ng/ml; Control: 1.8 ± 0.8 ng/ml; Obese: PCOS: 3.8 ± 0.3 ng/ml; Control: 2.0 ± 0.2 ng/ml	HDL-C, LDL-C, TG, SBP, DBP, nonHDL-C
Kargili (2010)	Turkery	Cross-section	168 vs. 52	PCOS: 25.7 ± 5.5 years; Control: 26.1 ± 5.4 years	PCOS: 26.8 ± 3.4 kg/m^2^; Control: 25.4 ± 2.8 kg/m^2^	–	–	HDL-C, LDL-C, TG, SBP, DBP, nonHDL-C
Ketel (2010)	Netherlands	Cross-section	Lean: 22 vs. 17 Obese: 18 vs. 13	Lean: PCOS: 28.6 ± 4.5 years; Control: 27.7 ± 5.3 years; Obese: PCOS: 30.3 ± 4.2 years; Control: 28.6 ± 5.3 years	Lean: PCOS: 22.0 ± 2.2 kg/m^2^; Control: 22.2 ± 1.7 kg/m^2^; Obese: PCOS: 36.2 ± 5.9 kg/m^2^; Control: 40.5 ± 7.0 kg/m^2^	Lean: PCOS: 0.78 ± 0.05; Control: 0.76 ± 0.03; Obese: PCOS: 0.84 ± 0.05; Control: 0.80 ± 0.01	Lean: PCOS: 7.0 ± 1.8 nmol/L; Control: 4.8 ± 1.2 nmol/L; Obese: PCOS: 7.7 ± 2.4 nmol/L; Control: 4.9 ± 1.7 nmol/L	HDL-C, LDL-C, TG, SBP, DBP, nonHDL-C
Legro (2001)	USA	Cross-section	Lean: 42 vs. 27; Obese: 153 vs. 35	Lean: PCOS: 25 ± 6 years; Control: 29 ± 7 years; Obese: PCOS: 28 ± 5 years; Control: 32 ± 7 years	Lean: PCOS: 23.1 ± 2.4 kg/m^2^; Control: 23.0 ± 1.8 kg/m^2^; Obese: PCOS: 37.0 ± 6.9 kg/m^2^; Control: 37.7 ± 6.4 kg/m^2^	Lean: PCOS: 0.76 ± 0.07; Control: 0.75 ± 0.06; Obese: PCOS: 0.85 ± 0.10; Control: 0.79 ± 0.06	Lean: PCOS: 2,553 ± 1,367 ng/ml; Control: 1,628 ± 734 ng/ml; Obese: PCOS: 2,476 ± 1,140 ng/ml; Control: 1,533 ± 720 ng/ml^a^	HDL-C, LDL-C, TG, SBP, DBP, nonHDL-C
Liang (2012)	Taiwan	Prospective	Lean: 110 vs. 50; Obese: 110 vs. 20	Lean: PCOS: 26.8 ± 5.1 years; Control: 28.1 ± 4.2 years Obese: PCOS: 27.0 ± 6.4 years; Control: 29.0 ± 5.1 years	Lean: PCOS: 20.6 ± 2.0 kg/m^2^; Control: 20.4 ± 2.0 kg/m^2^; Obese: PCOS: 31.1 ± 3.9 kg/m^2^; Control: 30.4 ± 3.7 kg/m^2^	Lean: PCOS: 0.79 ± 0.06; Control: 0.82 ± 0.14; Obese: PCOS: 0.89 ± 0.08; Control: 0.85 ± 0.08	–	HDL-C, LDL-C, TG, nonHDL-C
Long (2019)	China	Cross-section	387 vs. 150	PCOS: 27.0 ± 4.5 years; Control: 25.3 ± 2.2 years	PCOS: 25.4 ± 4.6 kg/m^2^; Control: 20.7 ± 2.6 kg/m^2^	PCOS: 0.86 ± 0.06; Control: 0.80 ± 0.07	–	HDL-C, LDL-C, TG, SBP, DBP, nonHDL-C
Luque-Ramirez (2007)	Spain	Cross-section	Lean: 11 vs. 8; Overweight: 13 vs. 4; Obese: 16 vs. 8	Lean: PCOS: 23.0 ± 5.4 years; Control: 24.8 ± 6.0 years; Overweight: PCOS: 23.6 ± 4.6 years; Control: 29.3 ± 10.3 years; Obese: PCOS: 26.3 ± 6.7 years; Control: 28.5 ± 5.8 years	Lean: PCOS: 22.2 ± 2.0 kg/m^2^; Control: 21.3 ± 1.3 kg/m^2^; Overweight: PCOS: 27.5 ± 1.8 kg/m^2^; Control: 27.4 ± 1.5 kg/m^2^ Obese: PCOS: 35.8 ± 3.9 kg/m^2^; Control: 35.5 ± 3.2 kg/m^2^	Lean: PCOS: 0.73 ± 0.06; Control: 0.73 ± 0.06; Overweight: PCOS: 0.79 ± 0.07; Control: 0.79 ± 0.04; Obese: PCOS: 0.88 ± 0.09; Control: 0.83 ± 0.08	PCOS: 12.7 ± 3.6 nmol/L; Control: 7.2 ± 2.2 nmol/L	HDL-C, LDL-C, TG, SBP, DBP, nonHDL-C
Macut (2008)	Serbia	Prospective	75 vs. 51	PCOS: 23.1 ± 5.1 years; Control: 24.6 ± 4.1 years	PCOS: 24.9 ± 4.7 kg/m^2^; Control: 23.7 ± 4.0 kg/m^2^	PCOS: 0.79 ± 0.06; Control: 0.77 ± 0.05	–	HDL-C, LDL-C, TG, nonHDL-C
Meyer (2005)	Australia	Retrospective	100 vs. 20	PCOS: 32.7 ± 1.8 years; Control: 33.2 ± 2.3 years	PCOS: 37.3 ± 2.43 kg/m^2^; Control: 36.7 ± 1.28 kg/m^2^	PCOS: 0.86 ± 0.01; Control: 0.84 ± 0.02	PCOS: 4.9 ± 0.3 mmol/L; Control: 3.6 ± 0.4 mmol/L^a^	HDL-C, LDL-C, TG, SBP, DBP, nonHDL-C
Moran (2009)	Australia	Cross-section	80 vs. 27	PCOS: 34.1 ± 6.9 years; Control: 33.8 ± 6.8 years	PCOS: 36.0 ± 6.6 kg/m^2^; Control: 37.4 ± 5.6 kg/m^2^	PCOS: 0.86 ± 0.08; Control: 0.84 ± 0.06	PCOS: 4.8 ± 0.3 µmol/L; Control: 3.4 ± 0.4 µmol/L^a^	HDL-C, LDL-C, TG, nonHDL-C
Ni (2009)	China	Retrospective	578 vs. 281	PCOS: 27.3 ± 3.7 years; Control: 28.3 ± 3.7 years	PCOS: 22.1 ± 3.7 kg/m^2^; Control: 22.2 ± 2.2 kg/m^2^	–	PCOS: 5.4 ± 2.5 µmol/L; Control: 4.4 ± 1.9 µmol/L^a^	TG, SBP, DBP
Oral (2009)	Turkey	Prospective	48 vs. 43	PCOS: 23.9 ± 3.3 years; Control: 24.2 ± 3.9 years	PCOS: 24.1 ± 2.9 kg/m^2^; Control: 24.0 ± 1.9 kg/m^2^	–	PCOS: 256.3 ± 59.5 µg/dl; Control: 246.5 ± 59.5 µg/dl^a^	HDL-C, LDL-C, TG, nonHDL-C
Orio (2004)	Italy	Prospective	30 vs. 30	PCOS: 22.2 ± 2.5 years; Control: 22.6 ± 2.3 years	PCOS: 22.4 ± 2.1 kg/m^2^; Control: 22.1 ± 1.8 kg/m^2^	PCOS: 0.77 ± 0.4; Control: 0.72 ± 0.3	PCOS: 4,535 ± 527 µmol/L; Control: 2,988 ± 311 µmol/L^a^	HDL-C, LDL-C, TG, SBP, DBP, nonHDL-C,
Philbois (2019)	Brazil	Retrospective	60 vs. 30	PCOS without obese: 28.5 ± 5.2 years; PCOS without obese: 30.2 ± 5.3 years; Control: 31.2 ± 6.6 years	PCOS without obese: 22.9 ± 1.6 kg/m^2^; PCOS with obese: 33.9 ± 2.4 kg/m^2^; Control: 23.5 ± 3 kg/m^2^	–	–	SBP, DBP
Rizzo (2011)	Italy	Prospective	350 vs. 90	PCOS: 24 ± 5 years; Control: 24 ± 3 years	PCOS: 27 ± 7 kg/m^2^; Control: 27 ± 4 kg/m^2^	–	–	HDL-C, LDL-C, TG, nonHDL-C
Sasaki (2011)	Japan	Prospective	54 vs. 24	PCOS: 30.2 ± 3.9 years; Control: 31.5 ± 4.4 years	PCOS: 24.3 ± 5.7 kg/m^2^; Control: 22.2 ± 3.4 kg/m^2^	–	–	HDL-C, LDL-C, TG, SBP, DBP, nonHDL-C
Shafiee (2020)	UK	Cross-section	34 vs. 34	PCOS: 31.8 ± 5.97 years; Control: 43.68 ± 13.12 years	PCOS: 29.28 ± 2.91 kg/m^2^; Control: 28.58 ± 2.62 kg/m^2^	PCOS: 0.88 ± 0.03; Control: 0.85 ± 0.02	–	HDL-C, LDL-C, TG, SBP, DBP, nonHDL-C
Shroff (2007)	USA	Prospective	24 vs. 24	PCOS: 32 ± 6.5 years; Control: 36 ± 7.2 years	PCOS: 36 ± 5.4 kg/m^2^; Control: 35 ± 3.3 kg/m^2^	PCOS: 0.85 ± 0.1; Control: 0.82 ± 0.1	–	HDL-C, LDL-C, TG
Soares (2009)	Brazil	Cross-section	40 vs. 50	PCOS: 24.5 ± 3.8 years; Control: 24.5 ± 5.1 years	PCOS: 22.7 ± 3.3 kg/m^2^; Control: 23.1 ± 3.2 kg/m^2^	–	–	HDL-C, LDL-C, TG, SBP, DBP, nonHDL-C
Tarkun (2004)	Turkey	Prospective	37 vs. 25	PCOS: 23.45 ± 4.3 years; Control: 24.4 ± 4.07 years	PCOS: 23.85 ± 3.26 kg/m^2^; Control: 22.9 ± 2.97 kg/m^2^	–	PCOS: 4.08 ± 2.3 ng/ml; Control: 2.89 ± 1.1 ng/ml	HDL-C, LDL-C, TG, nonHDL-C
Tiras (1999)	Turkey	Prospective	35 vs. 35	PCOS: 24.5 ± 6.0 years; Control: 23.6 ± 3.9 years	PCOS: 22.9 ± 4.2 kg/m^2^; Control: 22.0 ± 1.8 kg/m^2^	–	PCOS: 4.05 ± 3.14 ng/ml; Control: 2.48 ± 0.98 ng/ml	HDL-C, LDL-C, TG, nonHDL-C
Vryonidou (2005)	Greece	Prospective	75 vs. 55	PCOS: 23.9 ± 5.4 years; Control: 24.7 ± 5.3 years	PCOS: 27.3 ± 7.0 kg/m^2^; Control: 26.3 ± 7.7 kg/m^2^	PCOS: 0.79 ± 0.07; Control: 0.75 ± 0.04	PCOS: 8.12 ± 9.11 µmol/L; Control: 6.54 ± 8.59 µmol/L^a^	HDL-C, LDL-C, TG, SBP, nonHDL-C
Yildiz (2002)	Turkey	Prospective	59 vs. 23	PCOS: 22.9 ± 4.4 years; Control: 24.8 ± 4.2 years	PCOS: 23.0 ± 2.4 kg/m^2^; Control: 22.1 ± 2.2 kg/m^2^	PCOS: 0.76 ± 0.02; Control: 0.71 ± 0.02	PCOS: 9.4 ± 3.1 nmol/L; Control: 5.9 ± 1.7 nmol/L	HDL-C, TG, nonHDL-C

^a^ Represented that these studies measured the level of dehydroepiandrosterone sulfate, others measured androstenedione level. WHR: Waist-to-hip ratio; BMI: Body mass index; LDL-C: low-density lipoprotein cholesterol; TG: triglyceride; HDL-C: low high-density lipoprotein cholesterol; SBP: Systolic blood pressure; DBP: Diastolic blood pressure.

**Figure:**
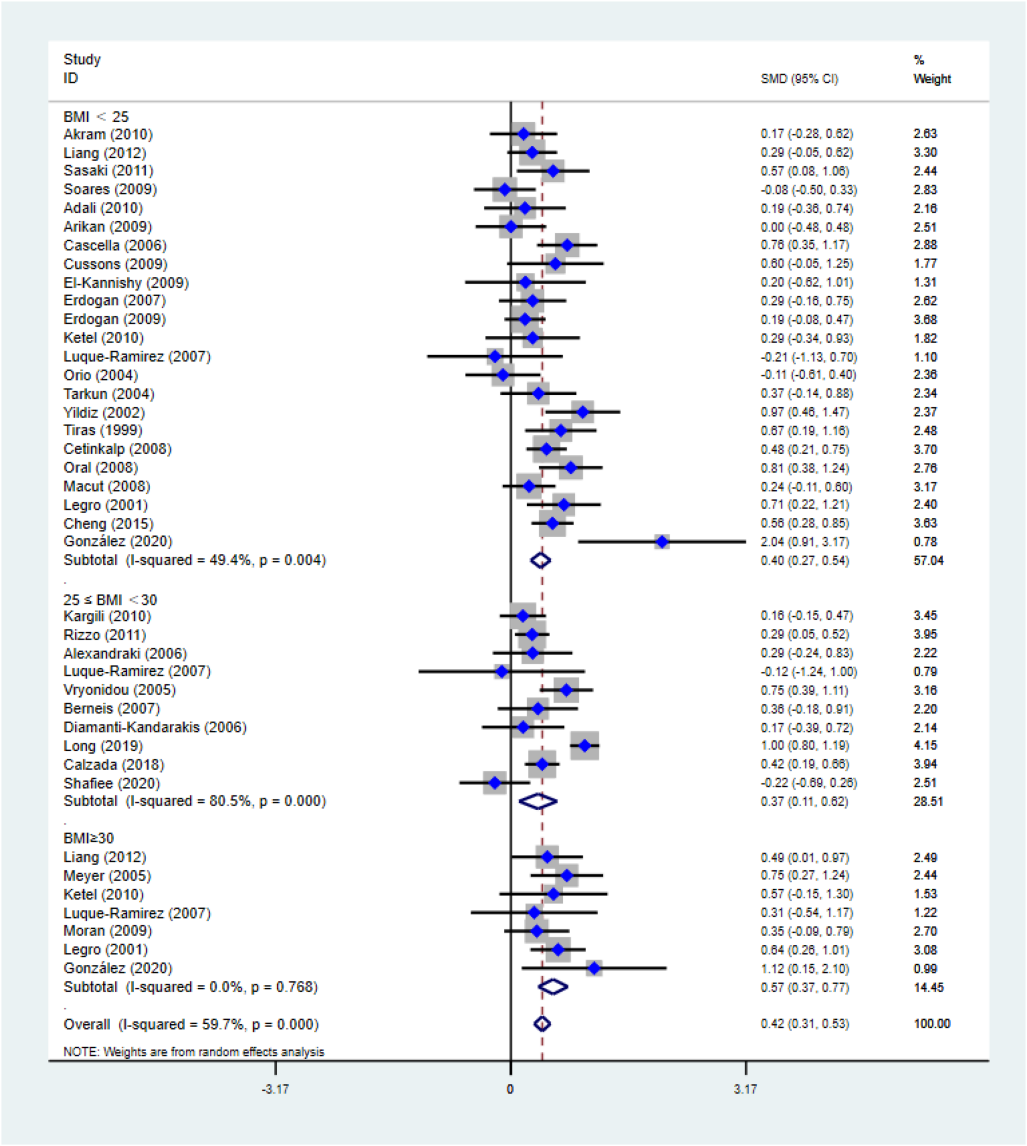


The authors apologize for this error and state that this does not change the scientific conclusions of the article in any way. The original article has been updated.

In the published article, there was an error in (Figure 4) as published. [We have revised Figure 4, in which we had included an incorrect study]. The corrected [Figure 4] and its caption **[Forest plot for comparison of non-high-density lipoprotein-cholesterol in polycystic ovary syndrome vs. control subjects. Studies are classified by different body mass index (BMI) categories (BMI < 25 kg/m^2^, BMI ≥ 30 kg/m^2^, and BMI 25–30 kg/m^2^).] appear above.

The authors apologize for this error and state that this does not change the scientific conclusions of the article in any way. The original article has been updated.

**Text Correction**

In the published article, there was two errors in the method.

1. According to the corrected figure, we revised the result of non-HDL-C].

An error was found in [Methods], [Study selection and criteria], [Page 2]. This sentence previously stated:

“[(4) articles published in languages other than English]” This sentence should be deleted.

2. [The selection criteria for the retrieved articles was unclear in the sixth information].

A correction has been made to [Methods], [Study selection and criteria], [Page 2]. This sentence previously stated:

“[6) each article had to conduct BMI matching]”

The corrected sentence appears below: 6) each article had to conduct BMI matching or the equal BMI (mean)

A correction has been made to [Results], [Lipid profiles], [Page 8]. This sentence previously stated:

“[As shown in Figure 4, non-HDL-C [SMD (95% CI): 0.42 (0.31, 0.52), *P* < 0.001] increased in reproductive-aged women with PCOS, with significant between-study heterogeneity. The subgroup analysis showed that non-HDL-C increased in women with PCOS at the three BMI levels, including BMI < 25 kg/m^2^ [SMD (95% CI): 0.40 (0.27, 0.54), *P* < 0.001], BMI ≥ 30 kg/m^2^ [MD (95% CI): 0.52 (0.33, 0.70), *P* < 0.001], and BMI 25–30 kg/m^2^ [MD (95% CI): 0.37 (0.11, 0.62), *P* = 0.005]. In this analysis, no publication bias was evident (asymmetry test *P* = 0.291).]”

The corrected sentence appears below:

“[As shown in Figure 4, non-HDL-C [SMD (95% CI): 0.42 (0.31, 0.53), *P* < 0.001] increased in reproductive-aged women with PCOS, with significant between-study heterogeneity. The subgroup analysis showed that non-HDL-C increased in women with PCOS at the three BMI levels, including BMI < 25 kg/m^2^ [SMD (95% CI): 0.40 (0.27, 0.54), *P* < 0.001], BMI ≥ 30 kg/m^2^ [MD (95% CI): 0.57 (0.37, 0.77), *P* < 0.001], and BMI 25–30 kg/m^2^ [MD (95% CI): 0.37 (0.11, 0.62), *P* = 0.005].]”

The authors apologize for this error and state that this does not change the scientific conclusions of the article in any way. The original article has been updated.

